# Production of non-natural 5-methylorsellinate-derived meroterpenoids in *Aspergillus oryzae*

**DOI:** 10.3762/bjoc.20.56

**Published:** 2024-03-20

**Authors:** Jia Tang, Yixiang Zhang, Yudai Matsuda

**Affiliations:** 1 Department of Chemistry, City University of Hong Kong, Tat Chee Avenue, Kowloon, Hong Kong SAR, China

**Keywords:** biosynthesis, meroterpenoids, natural products, pathway engineering, terpene cyclases

## Abstract

Fungal meroterpenoids are diverse structurally intriguing molecules with various biological properties. One large group within this compound class is derived from the aromatic precursor 3,5-dimethylorsellinic acid (DMOA). In this study, we constructed engineered metabolic pathways in the fungus *Aspergillus oryzae* to expand the molecular diversity of meroterpenoids. We employed the 5-methylorsellinic acid (5-MOA) synthase FncE and three additional biosynthetic enzymes for the formation of (6*R*,10′*R*)-epoxyfarnesyl-5-MOA methyl ester, which served as a non-native substrate for four terpene cyclases from DMOA-derived meroterpenoid pathways. As a result, we successfully generated six unnatural 5-MOA-derived meroterpenoid species, demonstrating the effectiveness of our approach in the generation of structural analogues of meroterpenoids.

## Introduction

Meroterpenoids are a class of natural products partially biosynthesized from a terpenoid pathway; their non-terpenoid portions can be polyketides, indole, or shikimate-derived compounds [1–3]. Their hybrid nature significantly contributes to their structural diversity and wide range of biological activities. Although meroterpenoids are found ubiquitously in nature, as both primary and secondary metabolites, filamentous fungi stand out as the most prolific producers of meroterpenoids [1–3]. Representative fungal meroterpenoids of medicinal importance include pyripyropene A, a cholesterol acyltransferase inhibitor [4]; fumagillin, an antimicrobial agent [5]; and mycophenolic acid, a strong inosine 5-monophosphate dehydrogenase inhibitor [6]. The biosynthesis of fungal meroterpenoids has garnered interest in the organic chemistry field due to their structural complexity and associated intriguing enzymatic reactions and has thus been extensively researched for over a decade, providing a general understanding of their biosynthesis [7–8].

**Figure 1:**
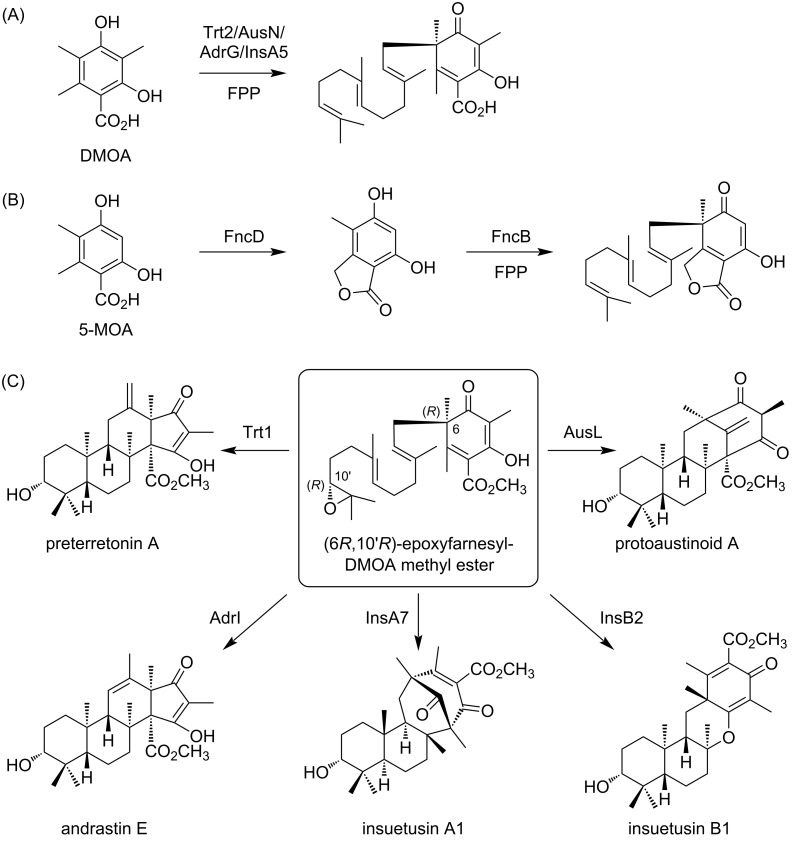
Biosynthesis of selected fungal meroterpenoids. (A, B) Dearomatizing prenylation reactions in the biosynthesis of (A) 3,5-dimethylorsellinic acid (DMOA)- and (B) 5-methylorsellinic acid (5-MOA)-derived meroterpenoids. (C) Reactions catalyzed by the terpene cyclases involved in DMOA-derived meroterpenoid pathways.

Polyketide–terpenoid hybrids are among the largest families of meroterpenoids. Orsellinic acid, an aromatic polyketide, and its analogues have been commonly identified as polyketide components in fungal meroterpenoids. Notably, 3,5-dimethylorsellinic acid (DMOA) serves as a precursor for a wide array of structurally diverse meroterpenoid species [7–8]. The fully substituted nature of DMOA leads to dearomatizing prenylation during the biosynthesis of DMOA-derived meroterpenoids (Figure 1A), facilitating the rearrangement reactions of the polyketide moiety, contributing to structural diversification [9–10]. By contrast, in the biosynthesis of meroterpenoids derived from orsellinic acid and 5-methylorsellinic acid (5-MOA), the prenylation reaction typically occurs at the non-substituted carbon atom and thus preserves the aromaticity of the polyketide portion [11–14]. One exception has been found in funiculolide biosynthesis, in which a 5-MOA-derived phthalide undergoes dearomatizing prenylation catalyzed by the UbiA-like prenyltransferase FncB (Figure 1B) [15]. In addition to prenyltransferases, transmembrane terpene cyclases play a key role in diversifying the structures of fungal meroterpenoids [16]. For example, (6*R*,10′*R*)-epoxyfarnesyl-DMOA methyl ester, a common intermediate with a linear terpenoid moiety, is known to be recognized by five different enzymes, namely Trt1, AusL, AdrI, InsA7, and InsB2, resulting in conversion into distinct cyclized products (Figure 1C) [17–19]. In addition, a recent study has demonstrated that some of these transmembrane terpene cyclases can accept synthetic substrate analogues to yield several unnatural meroterpenoid molecules [20]. By mimicking nature’s strategy to synthesize diverse meroterpenoids, we can access meroterpenoids that have not yet been reported.

In this study, we aimed to expand the structural diversity of meroterpenoids through artificial pathway construction. To achieve this, we constructed a series of artificial metabolic pathways by harnessing genes from the funiculolide biosynthetic pathway and those from DMOA-derived meroterpenoid pathways, successfully yielding six unnatural 5-MOA-derived meroterpenoid species.

## Results and Discussion

**Figure 2:**
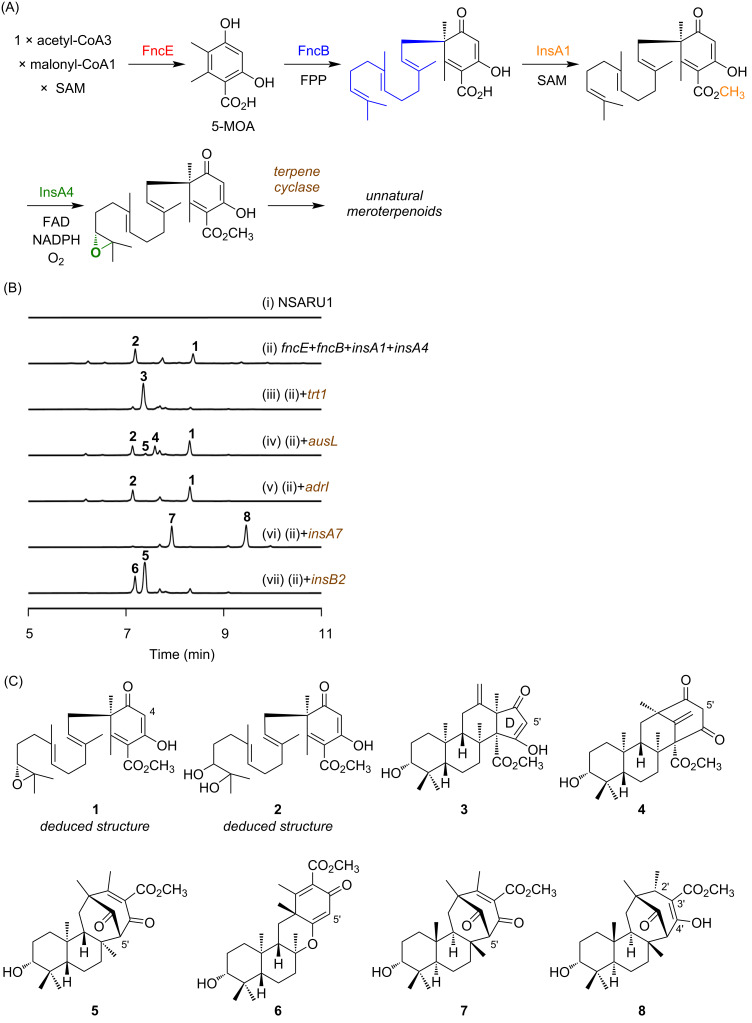
Generation of unnatural 5-MOA-derived meroterpenoids. (A) Working concept to synthesize unnatural 5-MOA-derived meroterpenoids. SAM: *S*-adenosyl-ʟ-methionine; FPP: farnesyl pyrophosphate; FAD: flavin adenine dinucleotide; NADPH: nicotinamide adenine dinucleotide phosphate. (B) HPLC profiles of the metabolites from *Aspergillus oryzae* transformants. The chromatograms were extracted at 254 nm. (C) Structures of metabolites detected or isolated in this study. Note that the structures of **1** and **2** were deduced based on their HRMS spectra and the predicted biosynthetic pathway.

To synthesize unnatural meroterpenoid molecules, we sought to generate a series of 5-MOA-derived meroterpenoids by utilizing the terpene cyclases responsible for DMOA-derived compounds. To achieve this goal, we first aimed to establish a production system for the 4-desmethyl analogue of (6*R*,10′*R*)-epoxyfarnesyl-DMOA methyl ester by utilizing the polyketide synthase FncE, the prenyltransferase FncB, the *O*-methyltransferase InsA1, and the FAD-dependent monooxygenase InsA4. In this engineered pathway, FncE first synthesizes 5-MOA, which then undergoes farnesylation by FncB, methyl ester formation by InsA1, and epoxidation of the terminal olefin in the farnesyl moiety by InsA4 (Figure 2A). Thus, we heterologously expressed the genes encoding these four enzymes in the *Aspergillus oryzae* NSARU1 strain [19]. Consequently, the *A. oryzae* transformant yielded two metabolites **1** and **2**, which were absent in the host strain (Figure 2B, traces i and ii). Although we were unable to isolate compounds **1** and **2** because of their instability, high-resolution mass spectrometry (HRMS) analysis revealed the molecular formula of **1** to be C_25_H_36_O_5_, corresponding to the 4-desmethyl form of (6*R*,10′*R*)-epoxyfarnesyl-DMOA methyl ester (Figure 2C). Furthermore, the molecular formula of **2** was determined to be C_25_H_38_O_6_, indicating that **2** is formed by the hydrolysis of the epoxide ring in **1** (Figure 2B). Taken together, this observation suggests that the desired production system was successfully established. Notably, although the native substrate of FncB is believed to be the phthalide form of 5-MOA (Figure 1B) [15], this result demonstrates that it can also efficiently accept 5-MOA as a substrate for dearomatizing farnesylation.

Subsequently, we introduced five terpene cyclase genes involved in DMOA-derived meroterpenoid biosynthesis, namely, *adrI*, *trt1*, *ausL*, *insA7*, and *insB2*, individually into the *A. oryzae* transformant that already expresses the four genes constructed earlier. We then analyzed the metabolites from the resulting transformants using high-performance liquid chromatography (HPLC), which revealed that all of the enzymes, except AdrI, accepted **1** and produced 5-MOA-derived meroterpenoids (Figure 2B, traces iii to vii). Since the transformation plasmid with *adrI* used in this study was constructed in our previous study, in which the product of AdrI was clearly detected [21], the inability of AdrI to yield a cyclized product is not likely to be caused by an inactive protein. The *trt1*-transformed strain produced a new compound **3** (molecular formula: C_25_H_36_O_5_). After large-scale cultivation, **3** was isolated and subjected to nuclear magnetic resonance (NMR) analysis, which suggested that **3** is the C-5′ desmethyl form of preterretonin A [17]. However, several missing signals in the ^13^C NMR spectrum, likely due to keto–enol tautomerization in the D-ring, hindered the complete structural determination of **3**. To overcome this challenge in structural determination, we obtained a single crystal of **3** and performed X-ray diffraction analysis, which unambiguously established the structure of **3** as 5′-desmethylpreterretonin A (Figure 2C and Figure S1 in Supporting Information File 1; CCDC: 2300693). The *A. oryzae* transformant with *ausL* yielded two products **4** and **5**. The major product **4** was identified as the C-5′ desmethyl analogue of protoaustinoid A and thus named 5′-desmethylprotoaustinoid A (Figure 2C) [17,22]. Meanwhile, the minor product **5** was determined as the C-5′ desmethyl form of the product from the K187A variant of AdrI, which was created during the in-depth functional analysis of terpene cyclases involved in DMOA-derived meroterpenoid biosynthesis [21], through NMR and single-crystal X-ray crystallographic analyses (Figure 2C and Figure S1 in Supporting Information File 1; CCDC: 2300694). Interestingly, compound **5** was also detected as a major product from the *A. oryzae* transformant harboring *insB2*, whereas the desmethyl analogue of the original InsB2 product [19], 5′-desmethylinsuetusin B1 (**6**) (Figure 2C), was only obtained as a minor product. Finally, the *A. oryzae* strain expressing *insA7* produced two major metabolites **7** and **8**. Compound **7** was determined to be the C-5′ desmethyl form of insuetusin A1 [19] using NMR and single-crystal X-ray diffraction analyses (Figure 2C and Figure S1 in Supporting Information File 1; CCDC: 2300695) and was designated as 5′-demethylinsuetusin A1. Unlike compounds **3**–**7**, the molecular formula of **8** was determined to be C_25_H_38_O_5_, which corresponds to the hydrogenated form of **7**. NMR analysis revealed that the double bond at C-2′/C-3′ of **7** was reduced to a single bond in **8** and that **8** contained the enol functionality instead of the C-4′ carbonyl group (Figure 2C). It is unlikely that **8** is the direct product of InsA7; thus, we hypothesized that an endogenous enzyme in *A. oryzae* is responsible for the reduction, with an enoylreductase first reducing the C-2′/C-3′ double bond of **7** and the resulting product undergoing keto–enol tautomerization to form **8** (Figure S2, Supporting Information File 1). Incubation of **7** with the host *A. oryzae* strain indeed resulted in the formation of **8** (Figure S3, Supporting Information File 1), confirming that InsA7 is not involved in the enoylreduction.

**Figure 3:**
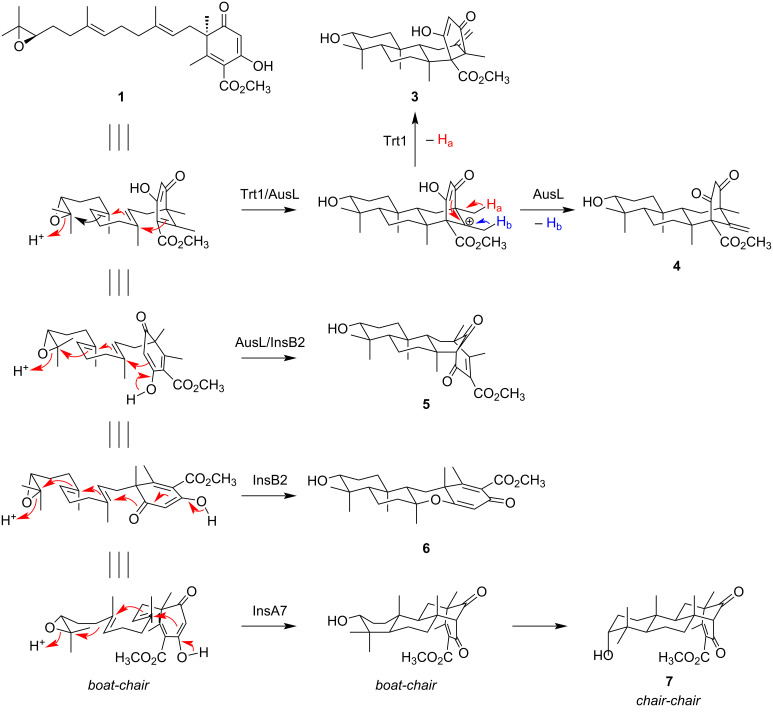
Biosynthetic mechanisms of the 5-MOA-derived meroterpenoids obtained in this study. In the reaction by InsA7, the cyclization should proceed via a pre-boat-chair conformation, and the conformational change after cyclization would afford the chair-chair conformation of **7** [19].

Our findings revealed that four of the five tested terpene cyclases could accept the desmethyl form of their native substrate, although the cyclization by AusL occurs less efficiently than by the other three enzymes. The cyclized products obtained in this study are all previously undescribed meroterpenoid species. The cyclization of **1** appears to occur in a manner similar to that of (6*R*,10′*R*)-epoxyfarnesyl-DMOA methyl ester (Figure 3). Although the major products from Trt1, AusL, and InsA7 are the C-5′ desmethyl forms of their original products, InsB2 displayed an altered cyclization preference toward the desmethyl analogue **1**, indicating that the missing methyl group significantly influences the folding mode of the substrate in InsB2. It remains unclear why AdrI was unable to accept **1**, but it is possible that the C-4 methyl group of (6*R*,10′*R*)-epoxyfarnesyl-DMOA methyl ester plays a critical role in substrate recognition by AdrI.

Finally, we evaluated the antibacterial activities of the compounds obtained in this study. As a result, only compound **3** exhibited weak activity against *Staphylococcus aureus* ATCC 6538 and *Bacillus cereus* with the minimum inhibitory concentration (MIC) of 500 µg/mL, respectively. Preterretonin A, the DMOA-derived counterpart of **3**, displayed weaker activity against the two bacterial strains and was not active at the concentration of 500 µg/mL. Thus, the lack of the C-5′ methyl group somehow contributes to the biological activity of **3**.

## Conclusion

We employed a pathway engineering approach to create a series of “unnatural” natural products by rationally combining the biosynthesis of DMOA- and 5-MOA-derived meroterpenoids. To the best of our knowledge, our study provides the first examples in which meroterpenoid species are generated via the direct dearomatizing prenylation of 5-MOA. Despite its simplicity, this strategy yielded six previously unreported meroterpenoids, demonstrating the effectiveness of our methodology in discovering new natural products. Given the increasing elucidation of fungal meroterpenoid pathways in recent years, similar approaches could be applied to other meroterpenoid biosynthetic processes, as reported in a recent study that yielded new DMOA-derived meroterpenoids with a monocyclic terpenoid moiety through pathway engineering [23]. Furthermore, the meroterpenoids produced in this study could be accepted by downstream enzymes in each pathway, further expanding the structural diversity of fungal meroterpenoids and potentially aiding the functional characterization of these tailoring enzymes.

## Supporting Information

File 1 Experimental details, analytical data, tables of primer sequences, constructed plasmids, and *A. oryzae* transformants and figures showing the X-ray crystal structures, the biosynthetic pathway, and NMR data and spectra.

## Data Availability

All data that supports the findings of this study is available in the published article and/or the supporting information to this article.
