# Supplementary material for: Production of non-natural 5-methylorsellinate-derived meroterpenoids in Aspergillus oryzae
Source: Beilstein J Org Chem. 2024 Mar 20;20:638–44. doi: 10.3762/bjoc.20.56 (PMC10964032; doi:10.3762/bjoc.20.56)
Supplement: File 1 — Experimental details, analytical data, tables of primer sequences, constructed plasmids, and A. oryzae transformants and figures showing the X-ray crystal structures, the biosynthetic pathway, and NMR data and spectra. [file Beilstein_J_Org_Chem-20-638-s001.pdf]

BEILSTEIN JOURNAL OF ORGANIC CHEMISTRY

Supporting Information

for

Production of non-natural 5-methylorsellinate-derived meroterpenoids in *Aspergillus oryzae*

Jia Tang, Yixiang Zhang and Yudai Matsuda

Beilstein J. Org. Chem. **2024,** *20,* 638–644. doi:10.3762/bjoc.20.56

Experimental details, analytical data, tables of primer sequences, constructed plasmids, and *A. oryzae* transformants and figures showing the X-ray crystal structures, the biosynthetic pathway, and NMR data and spectra

License and Terms: This is a supporting information file under the terms of the Creative Commons Attribution License (https://creativecommons.org/licenses/by/4.0). Please note that the reuse, redistribution and reproduction in particular requires that the author(s) and source are credited and that individual graphics may be subject to special legal provisions.
The license is subject to the *Beilstein Journal of Organic Chemistry* terms and conditions: (https://www.beilstein-journals.org/bjoc/terms)

Table of Contents

**Supplementary materials and methods**	**S2–S8**
**Supplementary Tables S1–S3**	**S9**
**Supplementary Figures S1–S45**	**S10–S34**
**Supplementary references**	**S35**

Supplementary materials and methods

General experimental procedures

Organic solvents were purchased from Anaqua (Hong Kong) Co. Ltd., and other chemicals were purchased from Wako Chemicals Ltd., Thermo Fisher Scientific, Sigma-Aldrich, or J&K Scientific Ltd., unless noted otherwise. Oligonucleotide primers (Table S1) were purchased from Beijing Genomics Institute. PCR was performed using a T100™ Thermal Cycler (Bio-Rad Laboratories, Inc.) with Phanta Max Super-Fidelity DNA Polymerase (Vazyme Biotech Co., Ltd). Analytical HPLC was performed on a Dionex Ultimate 3000 UHPLC system (Thermo Scientific). Semipreparative HPLC was performed on a Waters 1525 Binary HPLC pump with a 2998 photodiode array detector (Waters Corporation), using a COSMOSIL 5C18-AR-II column (10 i.d. × 250 mm, Nacalai Tesque, Inc). Flash chromatography was performed using an Isolera Spektra One flash purification system (Biotage). NMR spectra were obtained at 600 MHz (^{1}H)/150 MHz (^{13}C) with a Bruker Ascend Avance III HD spectrometer and analyzed by TopSpin 4.1.3 software. Chemical shifts were recorded with reference to solvent signals (^{1}H NMR: $CDCl_3$ 7.26 ppm, CD_3OD 3.31 ppm; ^{13}C NMR: $CDCl_3$ 77.0 ppm, CD_3OD 49.0 ppm). HR-ESI-MS spectra were obtained with a SCIEX X500R Q-TOF mass spectrometer. Optical rotations were measured with a P-2000 Digital Polarimeter (JASCO Corporation). X-ray diffraction data were collected on a Bruker D8 Venture Photon II diffractometer.

Strains

Aspergillus oryzae NSARU1 (*niaD$^-$, sC$^-$, ΔargB, adeA$^-$, pyrG$^-$*) [1] was utilized as the fungal heterologous expression host. Standard DNA engineering was performed with *Escherichia coli* DH5α (Takara Bio Inc).

Construction of fungal transformation plasmids

The polyketide synthase gene *fncE* and the prenyltransferase gene *fncB,* along with the *amyB* promoter (*PamyB*) and the *amyB* terminator (*TamyB*), were amplified from the previously constructed pTAex3-based plasmids [2], and were then introduced into the pPyrG-HR vector [1] and the previously constructed plasmid pAdeA-HR-insA1+insA4 [1], respectively, using a ClonExpress Ultra One Step Cloning Kit (Vazyme Biotech Co., Ltd), to yield pPyrG-HR-fncE and pAdeA-HR-insA1+insA4+fncB.

The primers used in the study and the detailed methods of the plasmid constructions are provided in Table S1 and Table S2.

Fungal transformation

Transformation of *A. oryzae* was carried out by the previously reported protoplast–polyethylene glycol method [3] coupled with CRISPR-Cas9-guided homologous recombination [1,4,5]. First, *A. oryzae* NSARU1 was transformed with the two plasmids, pPyrG-HR-fncE and pAdeA-insA1+insA4+fncB, to yield the expression

system of *fncE*, *fncB*, *insA1*, and *insA4*. The four gene-expressing *A. oryzae* transformant was then transformed with a plasmid containing a terpene cyclase gene, pTAex3-HR-trt1, pTAex3-HR-ausL, pTAex3-HR-adrI, pTAex3-HR-insA7, or pTAex3-HR-insB2, which had been constructed in our previous work [6]. The transformants created in this study and the plasmids used for the transformation are given in Table S3.

HPLC analysis of metabolites derived from *A. oryzae* transformants

To analyze the metabolites produced by each *A. oryzae* transformant, the transformants were cultivated on a DPY agar plate [2% dextrin, 1% hipolypepton (Nihon Pharmaceutical Co., Ltd.), 0.5% yeast extract, 0.5% KH_2PO_4, 0.05% $MgSO_4{\cdot}7H_2O$, and 1.5% agar] for seven days at 30 °C. A small piece of fungal mycelia and agar was cut from the plate, soaked in ethyl acetate, and extracted using an ultrasonic bath. The ethyl acetate layer was transferred to a new tube, and the solvent was removed using nitrogen gas flow.

The residue was dissolved in methanol and analyzed by HPLC, with a solvent system of 20 mM formic acid (solvent A) and acetonitrile containing 20 mM formic acid (solvent B), at a flow rate of 0.4 mL/min and a column temperature of 40 °C, using a Kinetex 2.6 μm C_{18} 100 Å column (2.1 i.d. x 100 mm; Phenomenex). Separation was performed using a linear gradient from 10:90 (solvent B/solvent A) to 100:0 for 10 min, 100:0 for the following 3 min, and a linear gradient from 100:0 to 10:90 within the following 2.0 min, and then 10:90 for 2.5 min of equilibrium.

Isolation of each metabolite from *A. oryzae* transformants

To isolate each metabolite, *A. oryzae* transformants were cultivated on DPY agar plates (90 mm diameter; the volume of medium in one plate is approximately 20 mL) for seven days at 30 °C. The resulting fungal cultures, including agar medium, were crushed into small pieces, soaked in ethyl acetate, and extracted twice using an ultrasonic bath. After filtration, ethyl acetate was removed in vacuo. The resultant crude extract was fractionated by flash chromatography, and further purified by semipreparative HPLC. Purification methods for each compound are described in detail below.

Purification conditions for 5′-desmethylpreterretonin A (**3**):

The extract of the *A. oryzae* strain with *fncE*, *fncB*, *insA1*, *insA4*, and *trt1* cultivated on 50 DPY agar plates was subjected to flash chromatography and eluted stepwise using a dichloromethane:ethyl acetate gradient (100:0 to 0:100). Fractions that contained **3** were then purified by reverse-phase semipreparative HPLC (45% aqueous acetonitrile, 3.0 mL/min) to yield 18.1 mg of **3**.

Purification conditions for and 5′-desmethylprotoaustinoid A (**4**) and **5**:

The extract of the *A. oryzae* strain with *fncE*, *fncB*, *insA1*, *insA4*, and *ausL* cultivated on 100 DPY agar plates was subjected to flash chromatography and eluted stepwise using a dichloromethane:ethyl acetate gradient

(100:0 to 0:100). Fractions that contained **4** and **5** were then purified by reverse-phase semipreparative HPLC (40% aqueous acetonitrile, 3.0 mL/min) to yield 25.1 mg of **4** and 12.1 mg of **5**.

Purification conditions for 5′-desmethylinsuetusin B1 (**6**):

The extract of the *A. oryzae* strain with *fncE*, *fncB*, *insA1*, *insA4*, and *insB2* cultivated on 60 DPY agar plates was subjected to flash chromatography and eluted stepwise using a dichloromethane:ethyl acetate gradient (100:0 to 0:100). Fractions that contained **6** were then purified by reverse-phase semipreparative HPLC (45% aqueous acetonitrile, 3.0 mL/min) to yield 4.1 mg of **6**.

Purification conditions for 5′-desmethylinsuetusin A1 (**7**) and **8**:

The extract of the *A. oryzae* strain with *fncE*, *fncB*, *insA1*, *insA4*, and *insA7* cultivated on 40 DPY agar plates was subjected to flash chromatography and eluted stepwise using a dichloromethane:ethyl acetate gradient (100:0 to 0:100). Fractions that contained **7** were then purified by reverse-phase preparative HPLC (55% aqueous acetonitrile, 3.0 mL/min) to yield 45.6 mg of **7**. Fractions that contained **8** were then purified by reverse-phase semipreparative HPLC (70% aqueous acetonitrile, 3.0 mL/min) to yield 70.1 mg of **8**.

X-ray crystallographic analysis

Single crystal of **3** was grown in CH_3OH, whereas those of **5** and **7** were grown in CH_3OH/CH_2Cl_2 (1:2, v/v), by a slow evaporation process at room temperature. Single crystal X-ray diffraction measurements were performed on a Bruker D8 Venture diffractometer using Cu Kα radiation at 213 K (for **3**), 218 K (for **5**), or 243 K (for **7**). The data collection was performed with the APEX3 program, and cell refinement and data reduction were carried out using the SAINT program. The structures of **3**, **5**, and **7** were solved by direct method with the SHELXT program and refined using the SHELXL program. All non-hydrogen atoms were refined anisotropically, whereas hydrogen atoms were placed by geometrical calculations. The absolute configuration of **3**, **5**, and **7** was determined by the Flack parameters.

Structural determination of each isolated compound

Structural determination of 5′-desmethylpreterretonin A (**3**):

The molecular formula of **3** was established as $C_{25}H_{36}O_5$ by HR-MS analysis. The NMR spectra of **3** are similar to those of preterretonin A [7] and revealed that the A-, B-, and C-rings of **3** are identical to those of preterretonin A. However, signals corresponding to the D-ring are missing in the ^1H and ^{13}C NMR spectra of **3**, hampering the complete structural determination based on the NMR analysis. The structure of **3**, including its absolute configuration, was established by X-ray crystallographic analysis, confirming that **3** is the 5′-desmethyl form of preterretonin A.

Structural determination of 5′-desmethylprotoaustinoid A (**4**):

The molecular formula of **4** was established as $C_{25}H_{36}O_5$ by HR-MS analysis. The NMR spectra of **4** are highly similar to those of protoaustinoid A [7], except that the signal corresponding to the methyl group bound to C-5′ is missing in the NMR spectra of **4** and that a methylene carbon (δ_C 59.5, δ_H 3.83/3.33) was observed in replacement of the C-5′ methine of protoaustinoid A. These observations suggested that **4** is the 5′-desmethyl form of protoaustinoid A, which was confirmed by further analysis of 2D NMR spectra of **4**.

Structural determination of compound **5**:

The molecular formula of **5** was established as $C_{25}H_{36}O_5$ by HR-MS analysis. The NMR spectra of **5** are highly similar to those of the major product from the K187A variant of AdrI [6], except that the signal corresponding to the methyl group bound to C-5′ is missing in the NMR spectra of **5** and that a methine signal (δ_H 3.12) was observed in the ^{1}H NMR spectrum of **5**. Finally, X-ray crystallographic analysis confirmed that **5** is the 5′-desmethyl form of the aforementioned product from the AdrI variant.

Structural determination of 5′-desmethylinsuetusin B1 (**6**):

The molecular formula of **6** was established as $C_{25}H_{36}O_5$ by HR-MS analysis. The NMR spectra of **6** are highly similar to those of insuetusin B1 [1], except that the signal corresponding to the methyl group bound to C-5′ is missing in the NMR spectra of **6** and that an olefinic proton (δ_H 5.78) was observed in the ^{1}H NMR spectrum of **6**. These observations suggested that **6** is the 5′-desmethyl form of protoaustinoid A, which was confirmed by further analysis of 2D NMR spectra of **6**.

Structural determination of 5′-desmethylinsuetusin A1 (**7**):

The molecular formula of **7** was established as $C_{25}H_{36}O_5$ by HR-MS analysis. The NMR spectra of **7** are highly similar to those of insuetusin A1 [1], except that the signal corresponding to the methyl group bound to C-5′ is missing in the NMR spectra of **7** and that a methine signal (δ_H 2.79) was observed in the ^{1}H NMR spectrum of **7**. Finally, X-ray crystallographic analysis confirmed that **7** is the 5′-desmethyl form of insuetusin A1.

Structural determination of compound **8**:

The molecular formula of **8** was established as $C_{25}H_{38}O_5$ by HR-MS analysis. The NMR spectra of **8** are similar to those of **7**, and it was revealed that **8** possesses an identical terpenoid moiety to **7**. However, the signals corresponding to the α,β-unsaturated carbonyl system at C-2′–C-4′ of **7** are missing in the ^{13}C NMR spectrum of **8**, which instead revealed the presence of one methine at 38.9 ppm (C-2′) and two *sp*^{2} quaternary carbons at 105.1 ppm (C-3′) and 168.5 ppm (C-4′). In addition, the ^{1}H-^{1}H COSY spectrum revealed the spin systems of H-2′/H-8′. Furthermore, the HMBC correlations of an enolic proton (δ_H 12.41) to C-3′ and C-4′ elucidated that the double bond at C-2′/C-3′ of **7** was reduced to a single bond in **8** and that **8** contained the enol functionality at

the C-4′ position. Finally, the 2′*S* configuration was established based on the correlations of H-8′ (δ 1.25) and H-9/H11α.

Feeding experiment

To perform the bioconversion experiment with compound **7**, *A. oryzae* NSARU1 was initially cultivated in 5 mL of DPY medium (supplemented with 0.01% adenine, 0.2% uracil, and 0.5% uridine) at 30 ºC and 160 rpm for three days and then transferred to 25 mL of DPY medium (supplemented with 0.01% adenine, 0.2% uracil, and 0.5% uridine) containing 16.7 mg/L of **7**. After cultivation at 30 ºC and 160 rpm for three days, the medium and mycelia were separated by filtration. The medium was extracted with ethyl acetate. Meanwhile, the mycelia were soaked in acetone for 1 hour using an ultrasonic bath, and after filtration, acetone was removed using nitrogen gas flow. The broth and mycelial extracts were individually dissolved in methanol and analyzed by HPLC with 60% aqueous acetonitrile containing 20 mM formic acid at a flow rate of 1.0 mL/min and a column temperature of 40 °C, using an Accucore C18 column (4.6 i.d. x 100 mm; Thermo Scientific).

Evaluation of the antibacterial activities of the obtained compounds

The compounds obtained in this study were initially tested for their antimicrobial activity using the Bauer-Kirby method. Five bacterial strains (*Staphylococcus epidermidis* ATCC 12228, *Staphylococcus aureus* ATCC 6538, *Bacillus cereus*, *Streptococcus faecalis*, and *Escherichia coli* ATCC 10536) were cultivated in Luria-Bertani (LB) broth at 37 °C for 12 hours and then diluted to a concentration of $\approx 5 \times 10^5$ colony forming unit (CFU)/mL using Mueller Hinton broth (beef infusion solids 2 g/L, starch 1.5 g/L, casein hydrolysate 17.5 g/L). Subsequently, 180 μL of the prepared microbial-containing medium was spread onto LB plates. After the surfaces of the plates dried, a 5 mm filter paper impregnated with a continuous 2-fold dilution of compounds (from 20 to 0.625 mg/mL) was placed at a specific location on each plate. The LB plates were incubated at 37 °C for 24 hours. Ampicillin was used as a positive control.

For compound **3**, which exhibited positive activity against *Staphylococcus aureus* ATCC 6538 and *Bacillus cereus*, the broth microdilution method was employed to determine the minimum inhibitory concentration (MIC) using a sterile 96-well plate. The bacteria-containing medium was prepared using the same method described above and added to each well of the 96-well plate (100 μL/well). The microbial strain was then treated with a continuous 2-fold dilution of the compound (from 1,000 to 1.95 μg/mL). The 96-well plate was placed in an incubator at 37 °C for 12 hours. The MIC was defined as the lowest concentration at which no bacterial growth was observed. The antibacterial activity of preterretonin A, which was obtained in our previous study [6], was measured in the same manner as described for **3**.

Analytical data

5′-desmethylpreterretonin A (3). White crystal; $[\alpha]^{22.7}_{D}$ –12.1 (*c* 1.00, $CHCl_3$); for NMR data see Figure S4

to Figure S10; HRMS (ESI) *m/z*: $[M + H]^+$ Calcd for $C_{25}H_{37}O_5$ 417.2636; Found 417.2618 (error = 4.31 ppm).

*5'-Desmethylprotoaustinoid A (**4**).* White amorphous solid; $[\alpha]^{22.1}_D$ –1.4 (*c* 0.69, $CHCl_3$); for NMR data see Figure S11 to Figure S17; HRMS (ESI) *m/z*: $[M + H]^+$ Calcd for $C_{25}H_{37}O_5$ 417.2636; Found 417.2616 (error = 4.79 ppm).

*Compound **5**.* White crystal; $[\alpha]^{22.0}_D$ +35.9 (*c* 0.90, $CHCl_3$); for NMR data see Figure S25 to Figure S31; HRMS (ESI) *m/z*: $[M + H]^+$ Calcd for $C_{25}H_{37}O_5$ 417.2636; Found 417.2617 (error = 4.55 ppm).

*5'-Desmethylinsuetusin B1 (**6**).* White amorphous solid; $[\alpha]^{21.2}_D$ –115.2 (*c* 0.34, $CHCl_3$); for NMR data see Figure S18 to Figure S24; HRMS (ESI) *m/z*: $[M + H]^+$ Calcd for $C_{25}H_{37}O_5$ 417.2636; Found 417.2617 (error = 4.55 ppm).

*5'-Desmethylinsuetusin A1 (**7**).* White crystal; $[\alpha]^{21.9}_D$ +64.8 (*c* 1.00, $CHCl_3$); for NMR data see Figure S32 to Figure S38; HRMS (ESI) *m/z*: $[M + H]^+$ Calcd for $C_{25}H_{37}O_5$ 417.2636; Found 417.2618 (error = 4.31 ppm).

*Compound **8**.* White amorphous solid; $[\alpha]^{23.0}_D$ +7.6 (*c* 1.00, $CHCl_3$); for NMR data see Figure S39 to Figure S45; HRMS (ESI) *m/z*: $[M + H]^+$ Calcd for $C_{26}H_{39}O_5$ 419.2792; Found 419.2775 (error = 4.05 ppm).

*Crystallographic data for **3**.* $C_{26.25}H_{43.50}O_{7.50}$, M = 479.11, a = 12.3160(3) Å, b = 13.0208(3) Å, c = 16.4954(4) Å, α = 90°, β = 95.8220(10)°, γ = 90°, V = 2631.63(11) Å^3, T = 213(2) K, space group $P2_1$, Z = 4, μ(Cu Kα) = 0.710 mm^{-1}, 34 374 reflections measured, 10 280 independent reflections (R_{int} = 0.0594). The final R_1 values were 0.0530 ($I > 2\sigma(I)$). The final $wR(F^2)$ values were 0.1445 ($I > 2\sigma(I)$). The final R_1 values were 0.0559 (all data). The final $wR(F^2)$ values were 0.1487 (all data). The goodness of fit on F^2 was 1.036. Flack parameter 0.07(8). The crystallographic information file (CIF) for this crystal structure was submitted to The Cambridge Crystallographic Data Centre (CCDC) under reference number 2300693.

*Crystallographic data for **5**.* $C_{25}H_{36}O_5$, M = 416.54, a = 6.34090(10) Å, b = 14.4638(3) Å, c = 23.9774(5) Å, α = 90°, β = 90°, γ = 90°, V = 2199.05(7) Å^3, T = 218(2) K, space group $P4_3$, Z = 4, μ(Cu Kα) = 0.690 mm^{-1}, 28 957 reflections measured, 4345 independent reflections (R_{int} = 0.0932). The final R_1 values were 0.0546 ($I > 2\sigma(I)$). The final $wR(F^2)$ values were 0.1231 ($I > 2\sigma(I)$). The final R_1 values were 0.0413 (all data). The final $wR(F^2)$ values were 0.1280 (all data). The goodness of fit on F^2 was 1.058. Flack parameter 0.04(15). The crystallographic information file (CIF) for this crystal structure was submitted to The Cambridge Crystallographic Data Centre (CCDC) under reference number 2300694.

Crystallographic data for ***7***. $C_{26}H_{40}O_6$, $M = 448.58$, $a = 11.29(2)$ Å, $b = 7.411(12)$ Å, $c = 14.95(3)$ Å, $\alpha = 90°$, $\beta = 95.91(4)°$, $\gamma = 90°$, $V = 1244(4)$ Å^3, $T = 243(2)$ K, space group $P2_1$, $Z = 2$, μ(Cu Kα) = 0.673 mm^{-1}, 19 180 reflections measured, 5029 independent reflections ($R_{int} = 0.0572$). The final R_1 values were 0.0471 ($I > 2\sigma(I)$). The final $wR(F^2)$ values were 0.1143 ($I > 2\sigma(I)$). The final R_1 values were 0.0572 (all data). The final $wR(F^2)$ values were 0.1222 (all data). The goodness of fit on F^2 was 1.061. Flack parameter 0.3(3). The crystallographic information file (CIF) for this crystal structure was submitted to The Cambridge Crystallographic Data Centre (CCDC) under reference number 2300695.

Table S1. Primers used in this study.

Primer	Sequence (5′ to 3′)
fncE-F1	TCGAGCTCGGTACCCAGGATGGGCTCATTACCAGAG
fncE-R1	GAATGGAACGCATGGGTGTTGGTC
fncE-F2	CCATGCGTTCCATTCCAGACTGGTC
fncE-R2	CTACTACAGATCCCCAGTAGAACCAGCTAAAGTACAGC
InF-pAdeA_SpeI-F	TAGAGGATCTACTAGTCAAGAGCAGAATGTGAACG
InF-pAdeA_SpeI-R	AATCCATATGACTAGTGATACATGAGCTTCGGTG

Table S2. Plasmids constructed in this study and PCR conditions for the amplification of the inserts for the plasmid constructions.

Plasmid	Inserts	Primer 1	Primer 2	PCR Template	Vector
pPyrG-HR-fncE	1st half of *fncE*<br>2nd half of *fncE*	fncE-F1<br>fncE-F2	fncE-R1<br>fncE-R2	pTAex3-fncE<br>pTAex3-fncE	pPyrG-HR digested with *Sma*I
pAdeA-insA1+insA4+fncB	*PamyB-fncB-TamyB*	InF-pAdeA_SpeI-F	InF-pAdeA_SpeI-R	pTAex3-fncB	pAdeA-insA1+insA4 digested with *Spe*I

Table S3. *Aspergillus oryzae* transformants constructed in this study.

Strain	Host strain	Plasmids used for transformation
A. oryzae/fncE+fncB+insA1+insA4	*A. oryzae* NSAR1	pPyrG-HR-fncE, pAdeA-insA1+insA4+fncB
A. oryzae/fncE+fncB+insA1+insA4+adrI	*A. oryzae/fncE+fncB+insA1+insA4*	pTAex3-HR-adrI
A. oryzae/fncE+fncB+insA1+insA4+trt1	*A. oryzae/fncE+fncB+insA1+insA4*	pTAex3-HR-trt1
A. oryzae/fncE+fncB+insA1+insA4+ausL	*A. oryzae/fncE+fncB+insA1+insA4*	pTAex3-HR-ausL
A. oryzae/fncE+fncB+insA1+insA4+insA7	*A. oryzae/fncE+fncB+insA1+insA4*	pTAex3-HR-insA7
A. oryzae/fncE+fncB+insA1+insA4+insB2	*A. oryzae/fncE+fncB+insA1+insA4*	pTAex3-HR-insB2

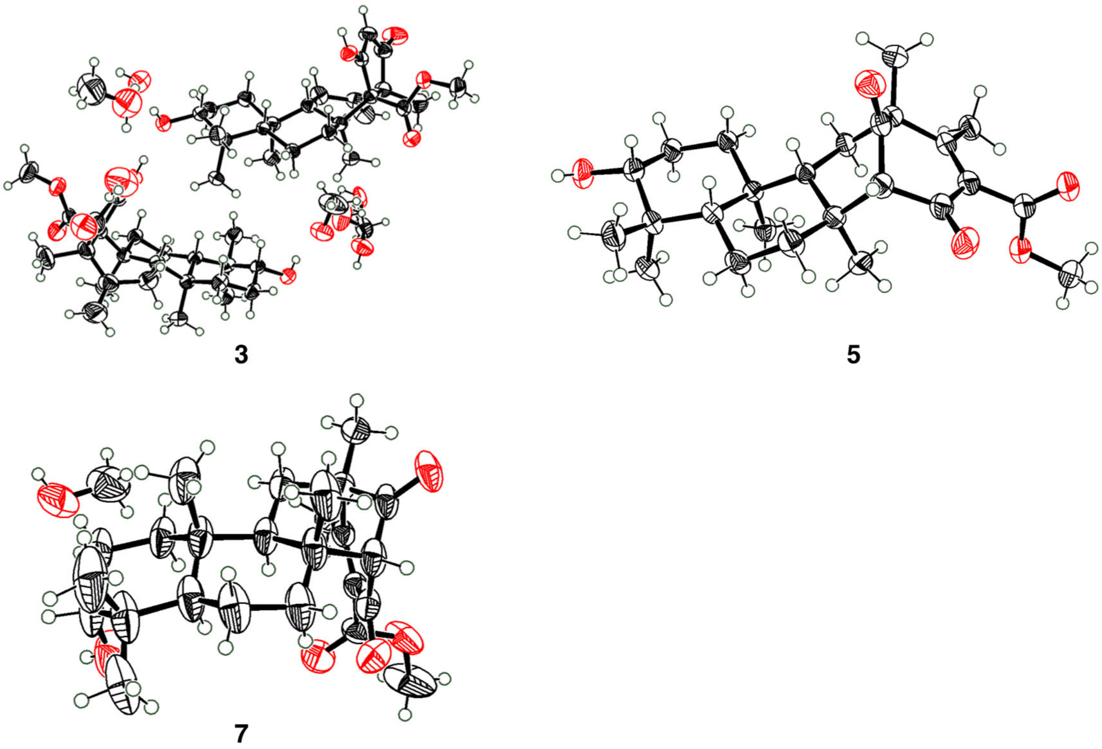

Figure S1. X-ray crystal structures of compounds **3**, **5**, and **7** (with 50% probability of thermal ellipsoid). The structures were drawn using ORTEP-3 for Windows version 2020.1 software [8].

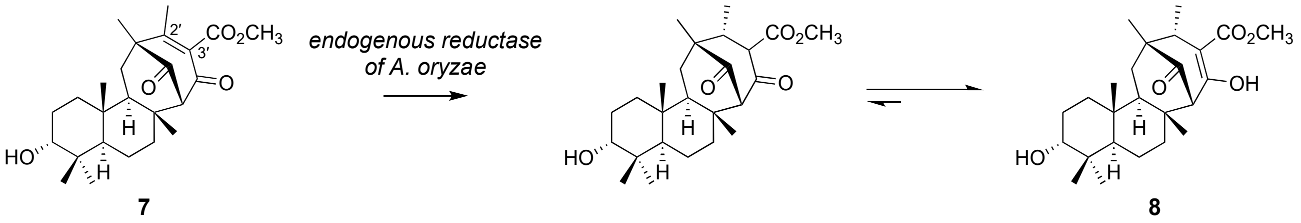

Figure S2. Predicted mechanism for the formation of **8**.

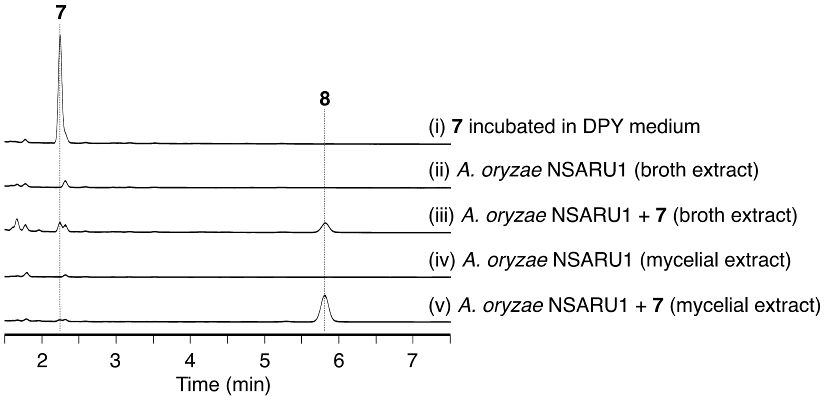

Figure S3. HPLC profile of (i) **7** incubated in DPY medium and the metabolites of *A. oryzae* NSARU1 cultivated (ii) without and (iii) with **7**. The chromatograms were extracted at 254 nm.

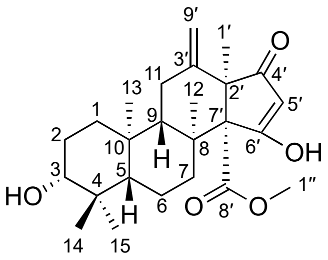

3

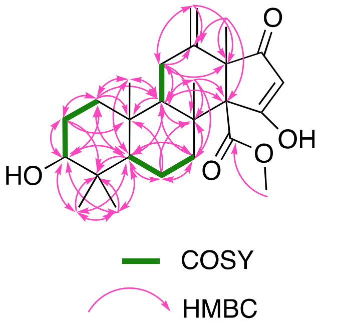

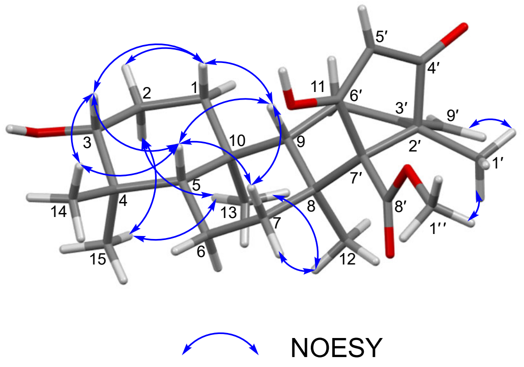

position	δ_C, type	δ_H, mult. (J in Hz)
1	38.8, CH_2	1.57 (α), dt (13.1, 3.6)
		0.86 (β), td (12.1, 3.5)
2	27.5, CH_2	1.62 (α), qd (12.1, 2.3)
		1.55 (β), m
3	79.6, CH	3.10, dd (11.6, 4.5)
4	39.8, C	
5	56.1, CH	0.80, dd (11.5, 2.3)
6	19.1, CH_2	1.51, m
7	35.4, CH_2	1.71 (α), dt (12.9, 3.3)
		2.61 (β), td (12.5, 3.9)
8	44.7, C	
9	51.0, CH	1.51, m
10	39.6, C	
11	30.9, CH_2	2.31, m
12	20.0, CH_3	1.36, s
13	15.8, CH_3	0.96, s
14	28.6, CH_3	0.96, s
15	16.1, CH_3	0.77, s
1′	19.2, CH_3	1.31, s
2′	57.4[a], C	
3′	149.6, C	
4′	n.d.	
5′	n.d.	n.d.
6′	n.d.	
7′	71.7, C	
8′	171.7, C	
9′	112.8, CH_2	5.08, d (1.9)
		5.05, d (1.9)
1″	51.7, CH_3	3.62, s

^{1}H NMR: 600 MHz, ^{13}C NMR: 150 MHz (in CD_3OD)

[a] Identified in the HMBC spectrum. n.d.: Not detected.

Figure S4. NMR data of 5′-desmethylpreterretonin A (**3**).

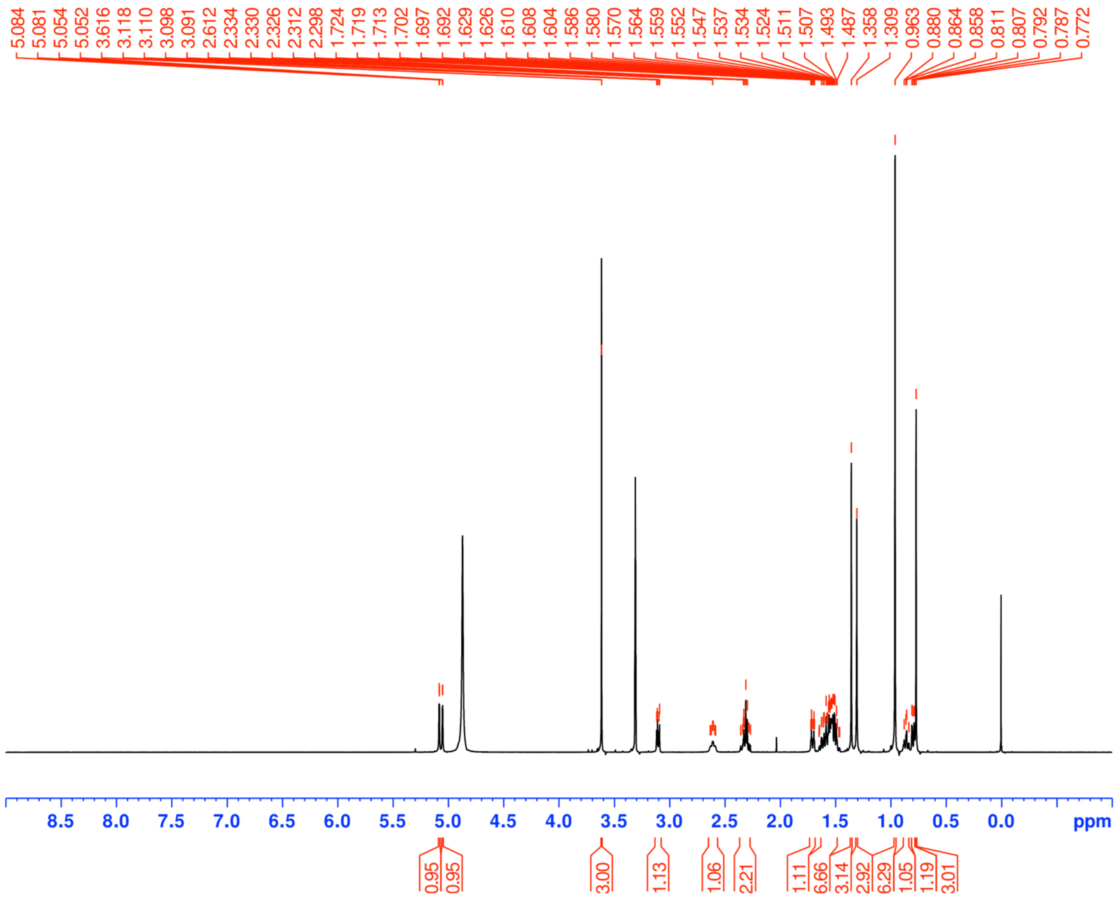

Figure S5. ^{1}H NMR spectrum of 5′-desmethylpreterretonin A (**3**) in CD_3OD at 600 MHz.

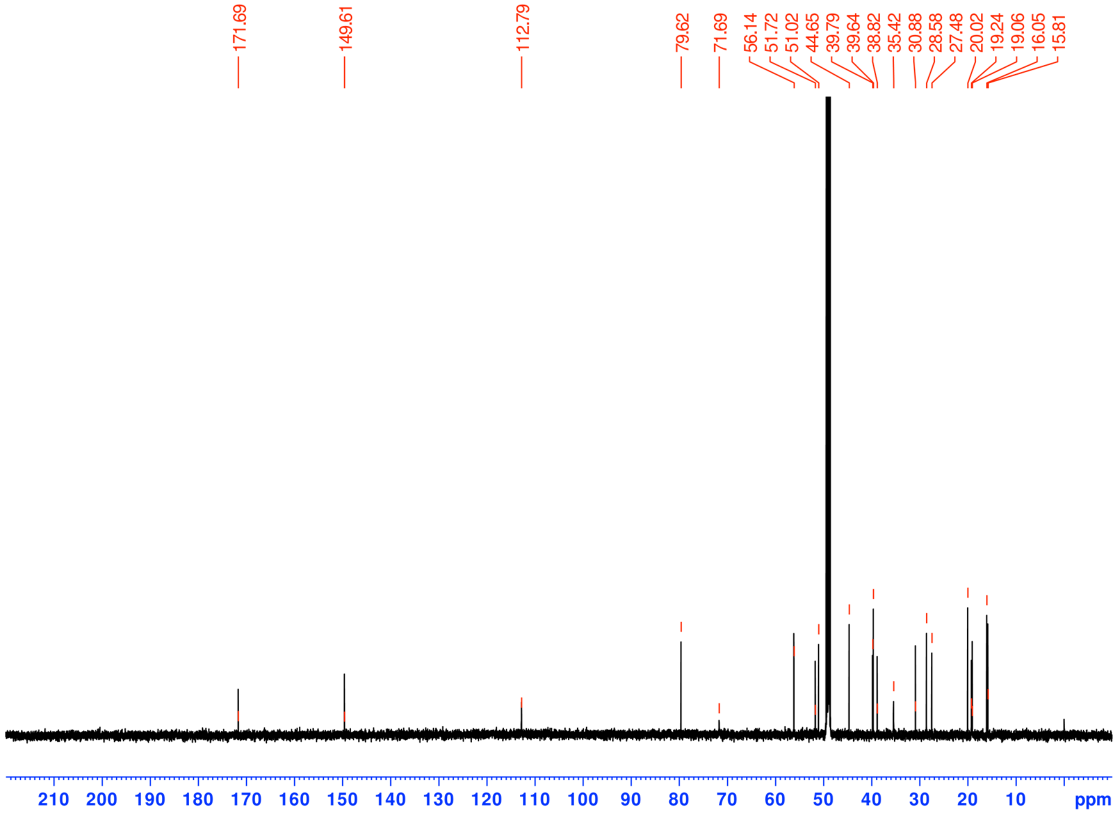

Figure S6. ^{13}C NMR spectrum of 5′-desmethylpreterretonin A (**3**) in CD_3OD at 150 MHz.

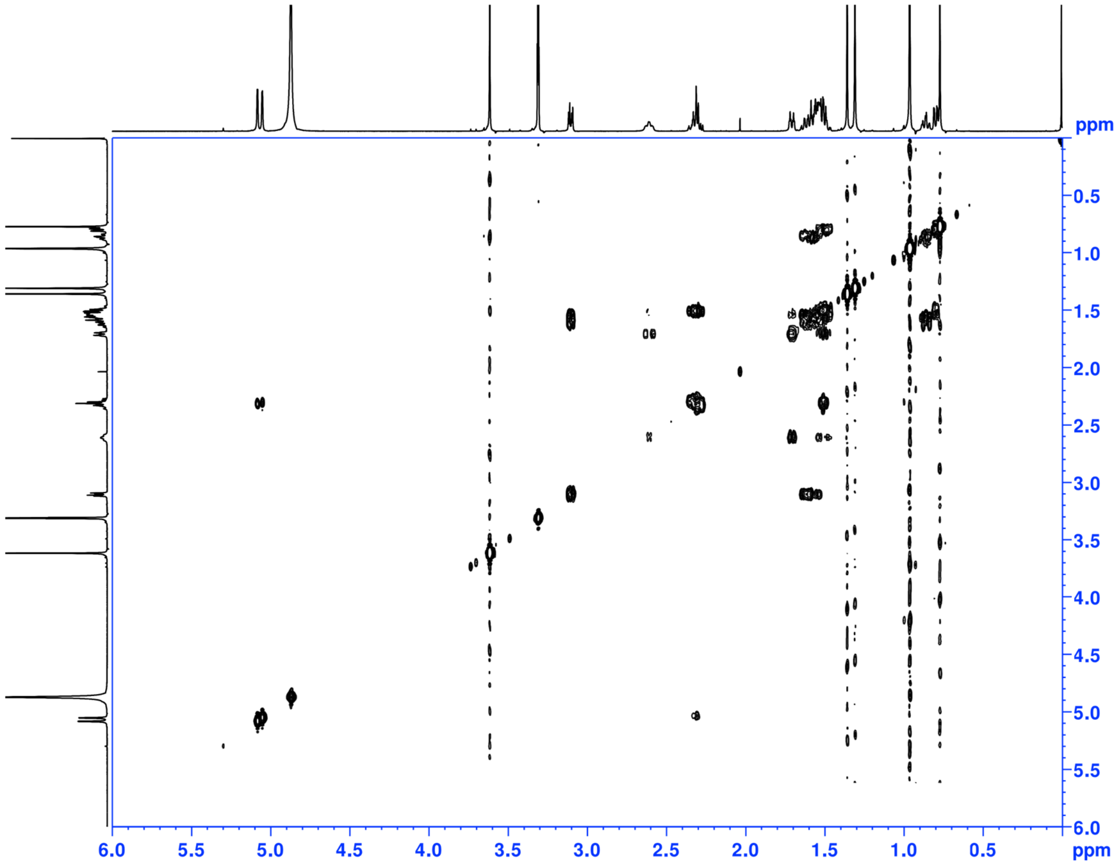

Figure S7. ^{1}H-^{1}H COSY spectrum of 5′-desmethylpreterretonin A (**3**) in CD_3OD.

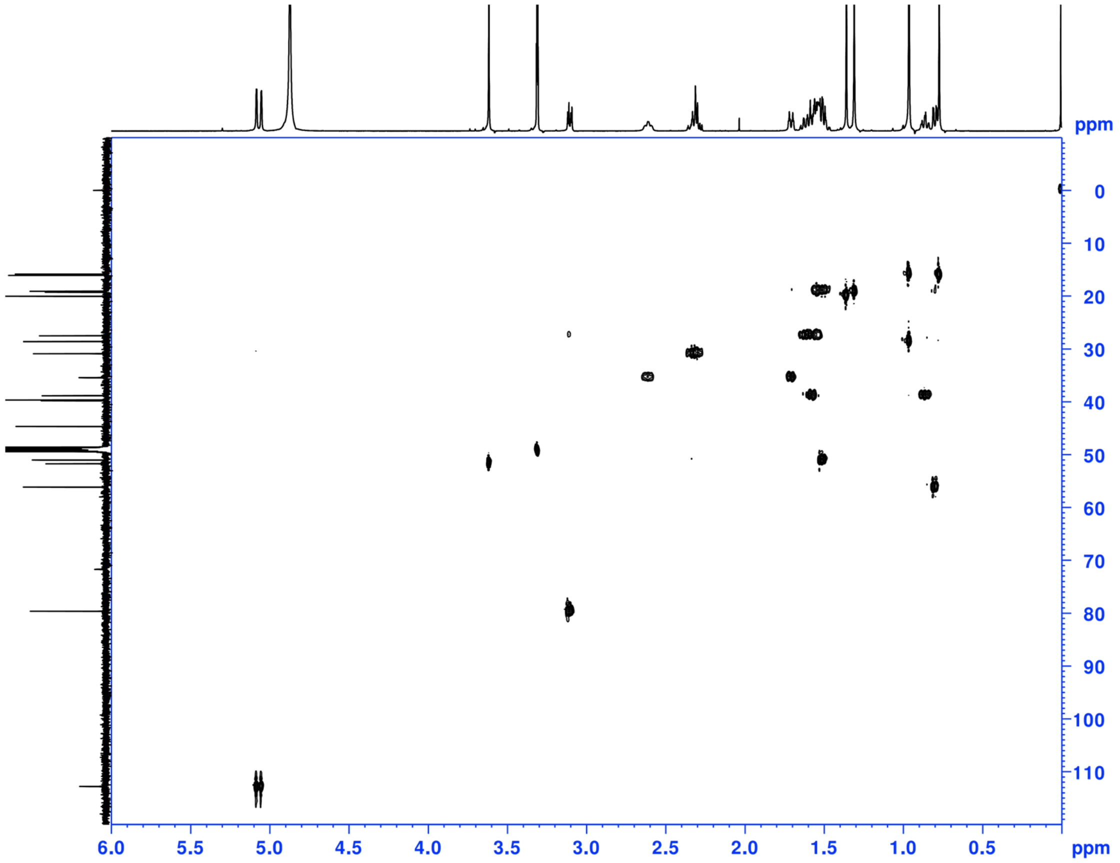

Figure S8. HSQC spectrum of 5′-desmethylpreterretonin A (**3**) in CD_3OD.

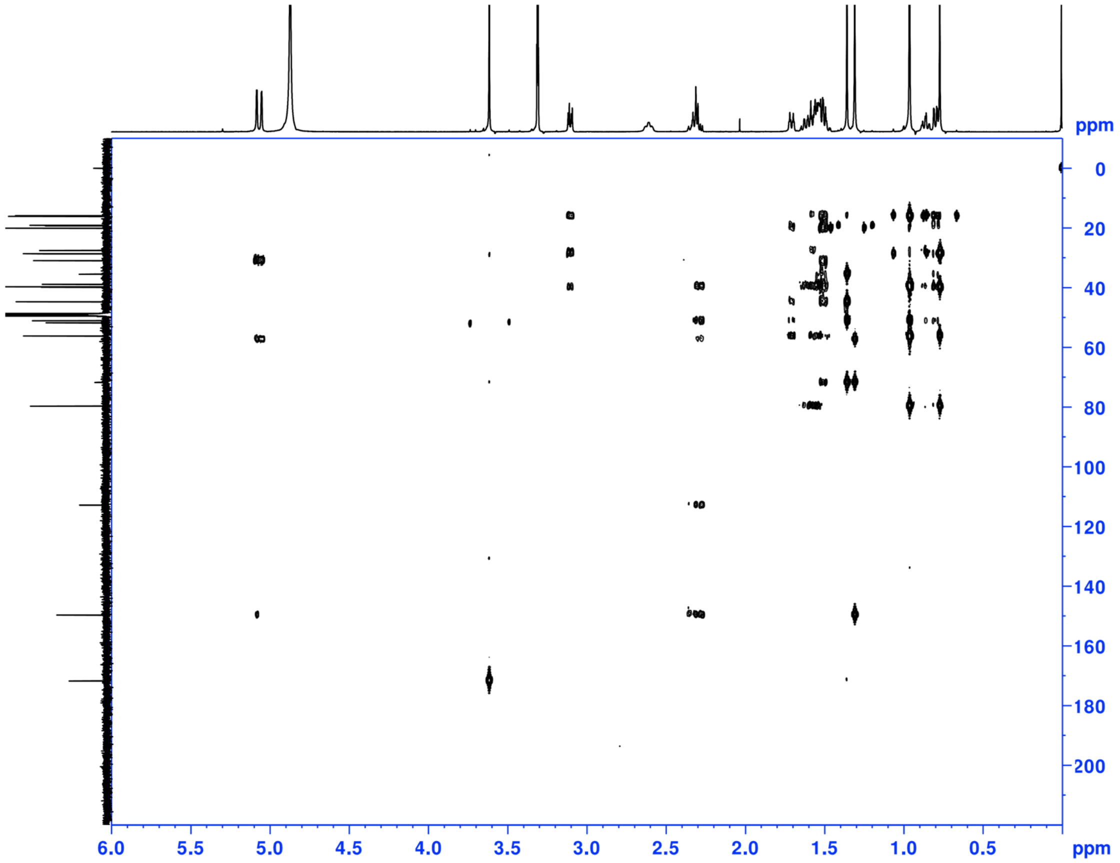

Figure S9. HMBC spectrum of 5′-desmethylpreterretonin A (**3**) in CD_3OD.

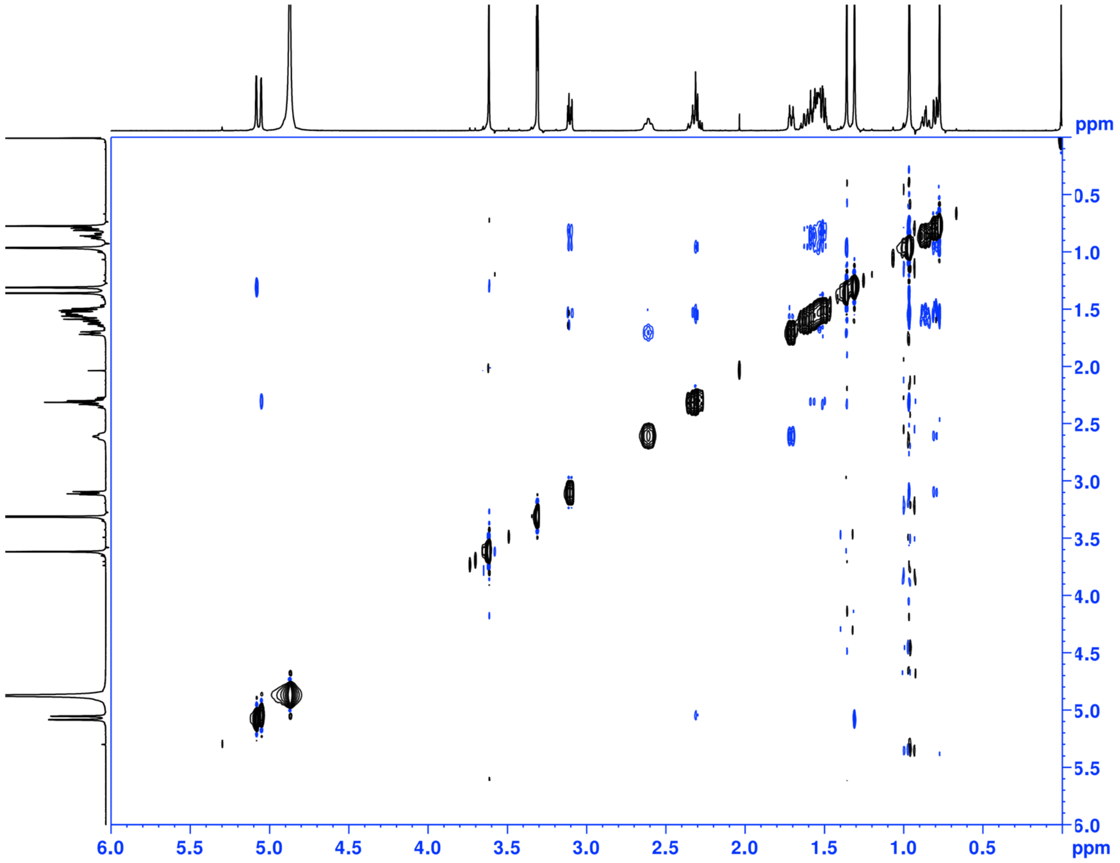

Figure S10. NOESY spectrum of 5′-desmethylpreterretonin A (**3**) in CD_3OD.

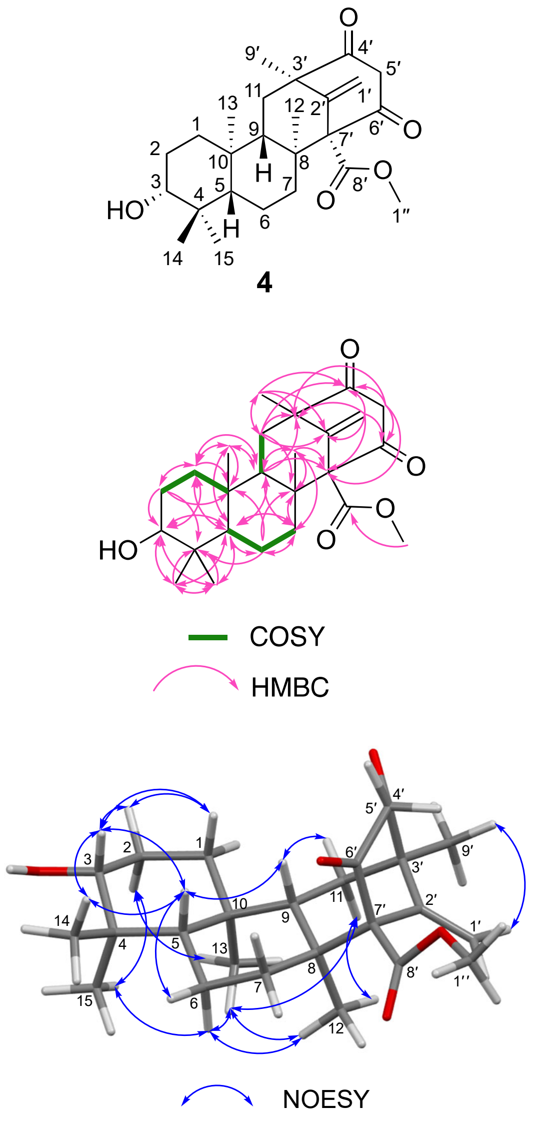

position	δ_C, type	δ_H, mult. (*J* in Hz)
1	38.2, CH_2	1.56 (α), m
		0.75 (β), m
2	26.9, CH_2	1.50 (α), qd (12.1, 3.4)
		1.58 (β), m
3	78.3, CH	3.13, dd (11.7, 4.5)
4	38.7, C	
5	54.5, CH	0.62, dd (12.1, 1.8)
6	18.2, CH_2	1.42 (α), qd (12.1, 4.7)
		1.60 (β), m
7	32.9, CH_2	2.15, m
8	46.9, C	
9	52.1, CH	0.80, dd (13.6, 3.2)
10	37.6, C	
11	37.6, CH_2	1.60 (α), m
		1.89 (β), dd (13.2, 3.3)
12	17.2, CH_3	1.21, s
13	15.8, CH_3	0.79, s
14	27.8, CH_3	0.94, s
15	15.4, CH_3	0.74, s
1′	112.8, CH_2	5.30, brs
		4.82, brs
2′	146.6, C	
3′	51.9, C	
4′	207.7, C	
5′	59.5, CH_2	3.83, d (17.2)
		3.33, d (17.2)
6′	203.1, C	
7′	73.2, C	
8′	169.1, C	
9′	20.9, CH_3	1.37, s
1″	51.9, CH_3	3.67, s

^{1}H NMR: 600 MHz, ^{13}C NMR: 150 MHz (in $CDCl_3$)

Figure S11. NMR data of 5′-desmethylprotoaustinoid A (**4**).

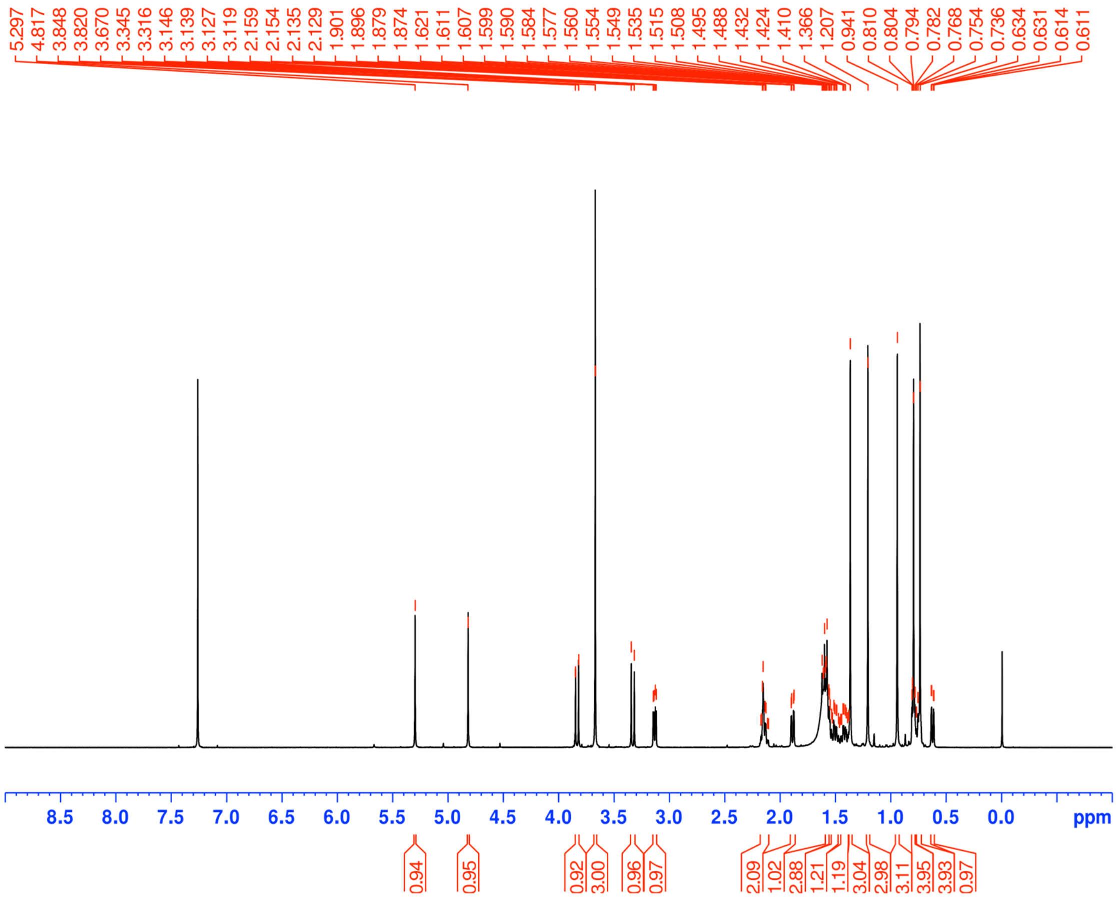

Figure S12. ^{1}H NMR spectrum of 5′-desmethylprotoaustinoid A (**4**) in $CDCl_3$ at 600 MHz.

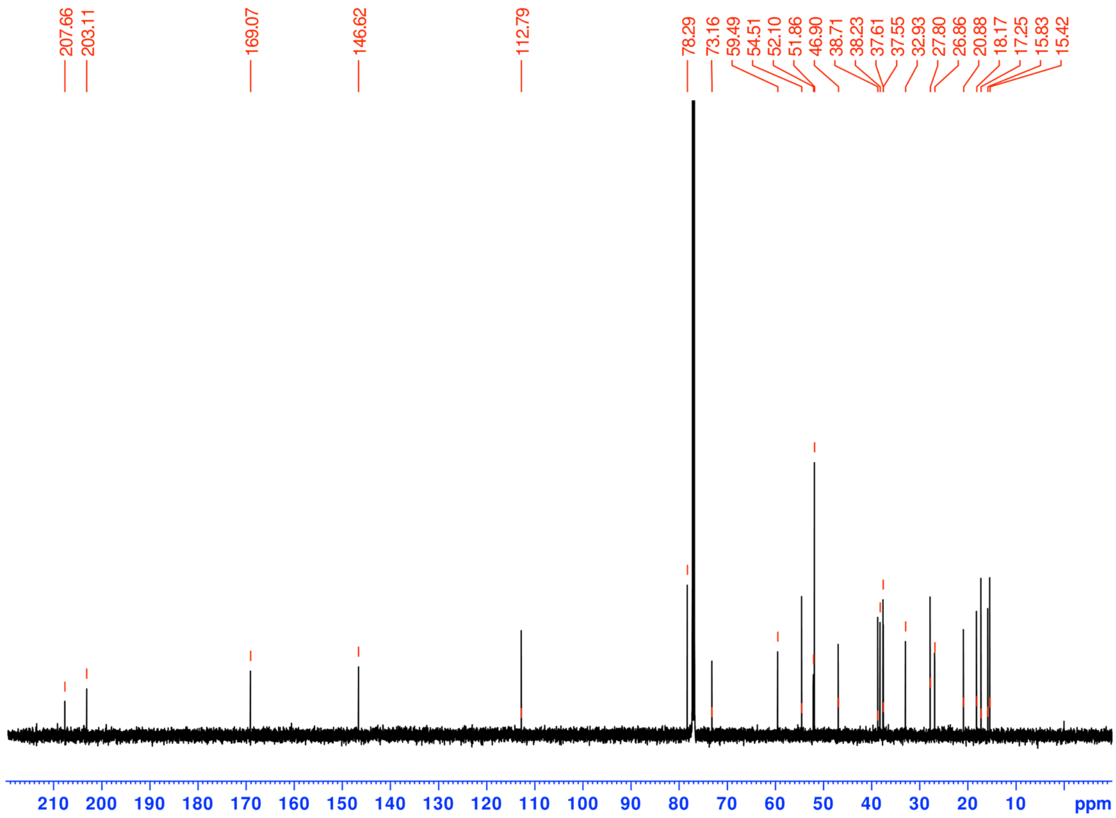

Figure S13. ^{13}C NMR spectrum of 5′-desmethylprotoaustinoid A (**4**) in $CDCl_3$ at 150 MHz.

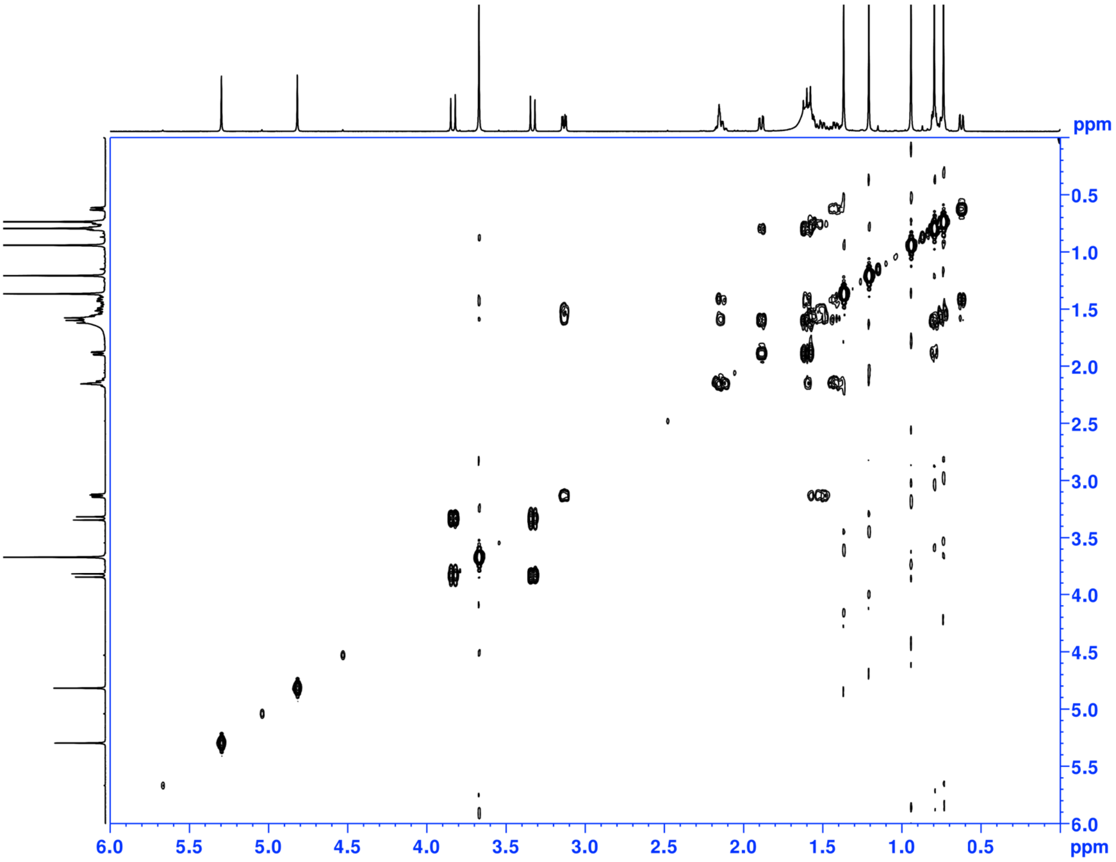

Figure S14. ^{1}H-^{1}H COSY spectrum of 5′-desmethylprotoaustinoid A (**4**) in $CDCl_3$.

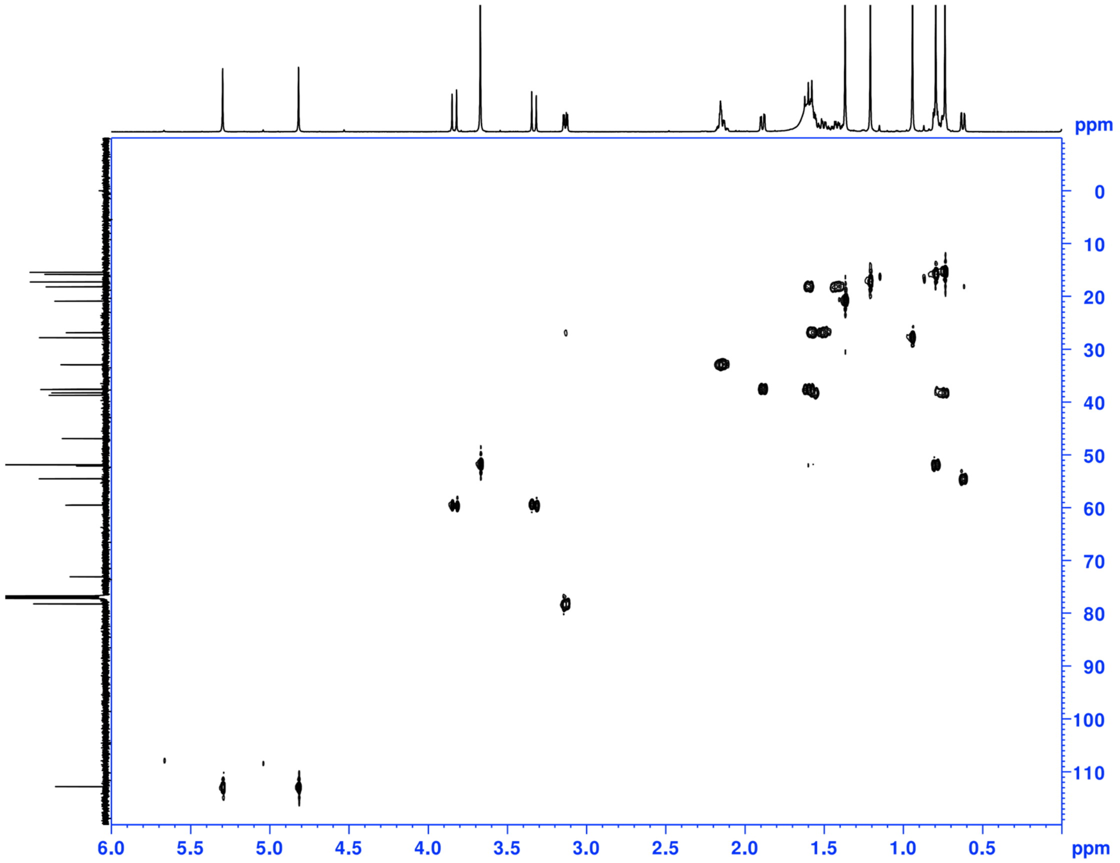

Figure S15. HSQC spectrum of 5′-desmethylprotoaustinoid A (**4**) in $CDCl_3$.

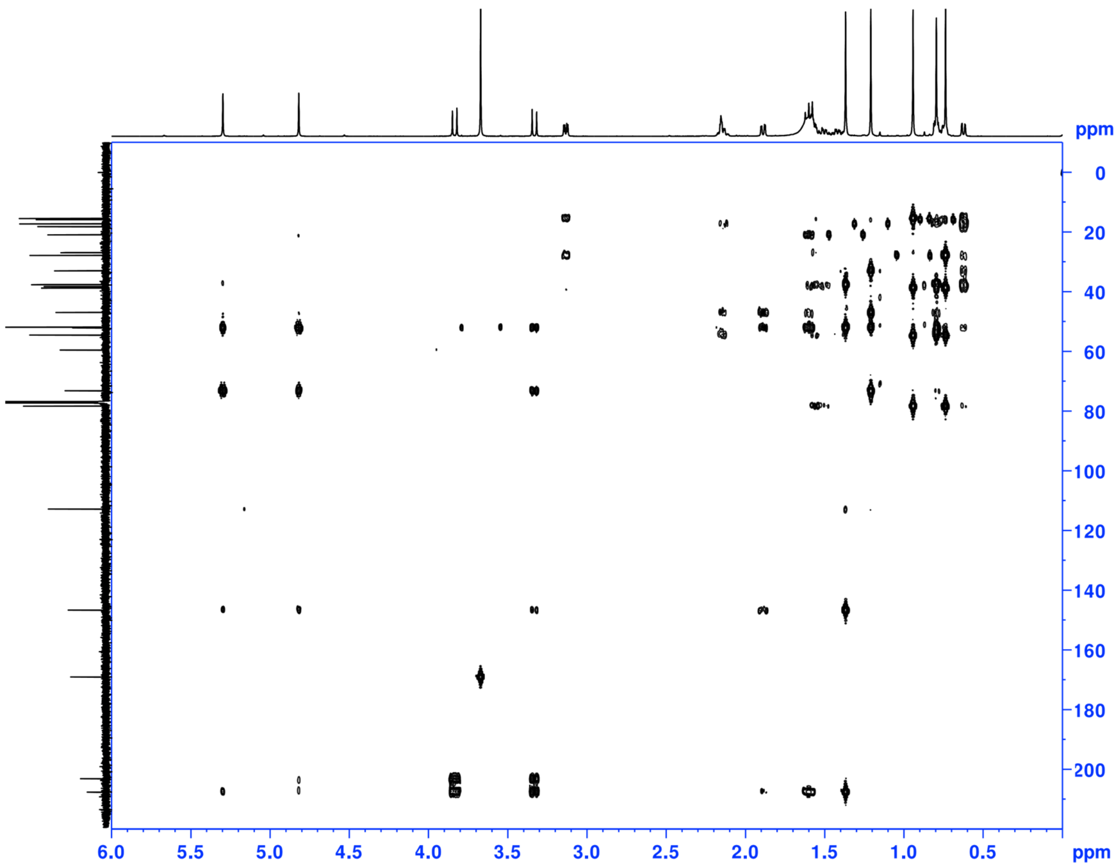

Figure S16. HMBC spectrum of 5′-desmethylprotoaustinoid A (**4**) in $CDCl_3$.

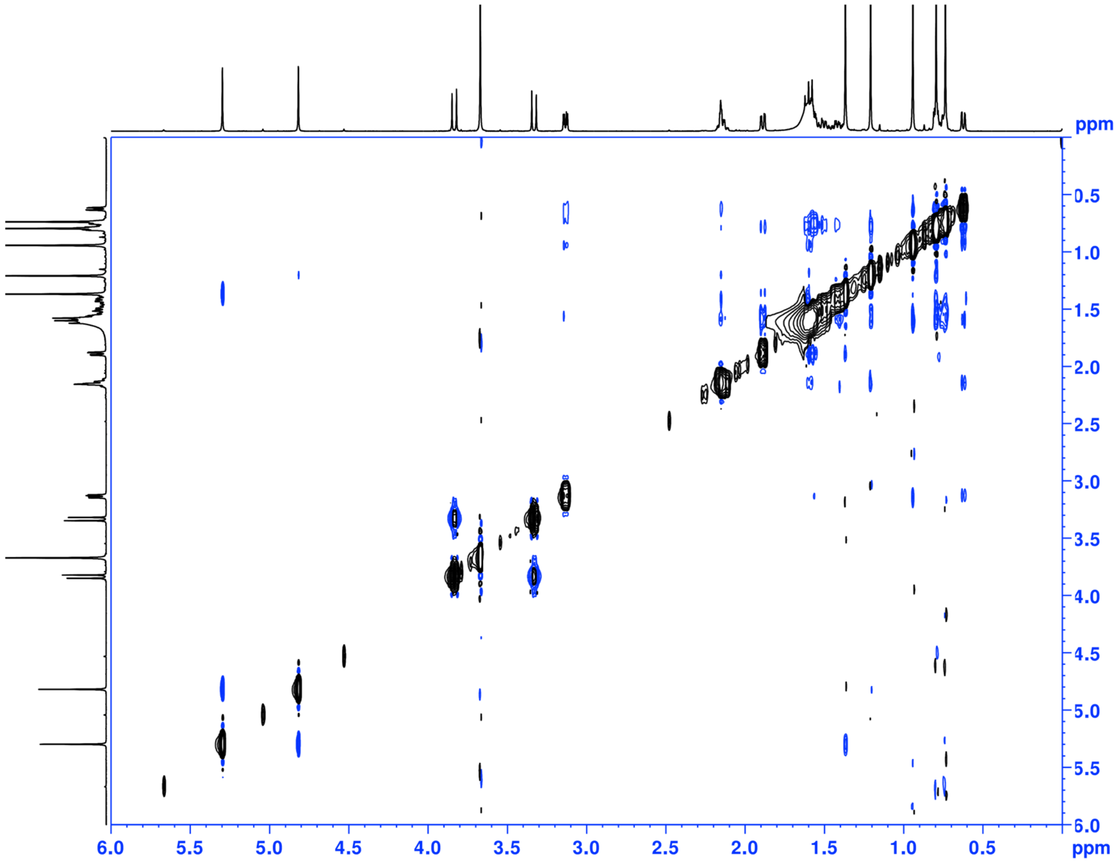

Figure S17. NOESY spectrum of 5′-desmethylprotoaustinoid A (**4**) in $CDCl_3$.

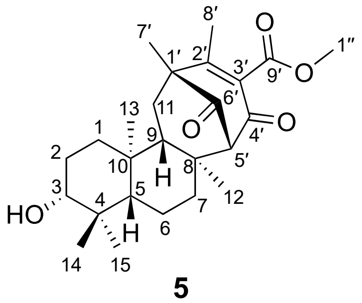

5

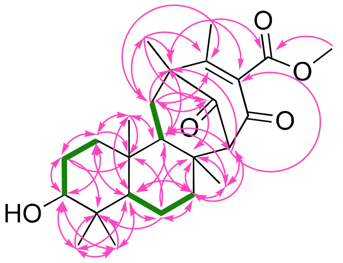

COSY

HMBC

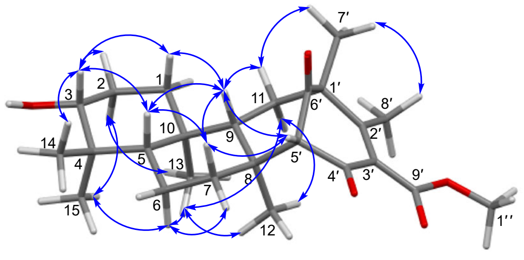

NOESY

position	δ_C, type	δ_H, mult. (*J* in Hz)
1	37.9, CH_2	1.58 (α), m
		0.94 (β), m
2	26.9, CH_2	1.58 (α), m
		1.65 (β), m
3	78.2, CH	3.18, dd (11.3, 4.6)
4	38.9, C	
5	55.3, CH	0.85, dd (12.5, 2.3)
6	18.7, CH_2	1.49 (α), qd (12.9, 2.0)
		1.59 (β), m
7	45.6, CH_2	1.89 (α), m
		1.46 (β), td (13.5, 3.3)
8	42.9, C	
9	55.8, CH	1.21, dd (13.8, 3.3)
10	37.5, C	
11	35.2, CH_2	2.16 (α), t (13.6)
		1.75 (β), dd (13.4, 3.4)
12	18.8, CH_3	1.15, s
13	16.7, CH_3	0.84, s
14	27.7, CH_3	0.95, s
15	15.2, CH_3	0.75, s
1′	49.3, C	
2′	163.1, C	
3′	131.7, C	
4′	192.9, C	
5′	74.8, CH	3.12, s
6′	207.4, C	
7′	19.9, CH_3	1.26, s
8′	17.6, CH_3	2.03, s
9′	166.5, C	
1″	52.5, CH_3	3.83, s

^{1}H NMR: 600 MHz, ^{13}C NMR: 150 MHz (in $CDCl_3$)

Figure S18. NMR data of **5**.

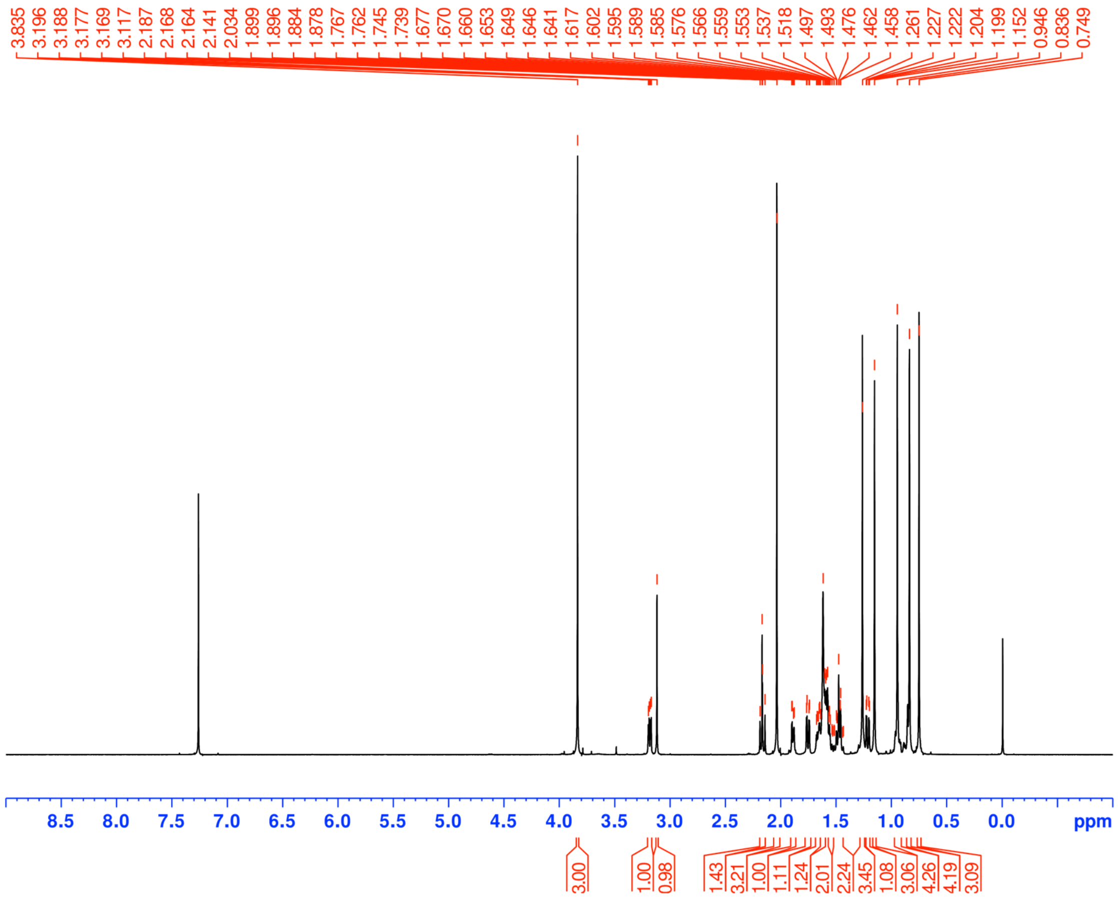

Figure S19. ^{1}H NMR spectrum of **5** in $CDCl_3$ at 600 MHz.

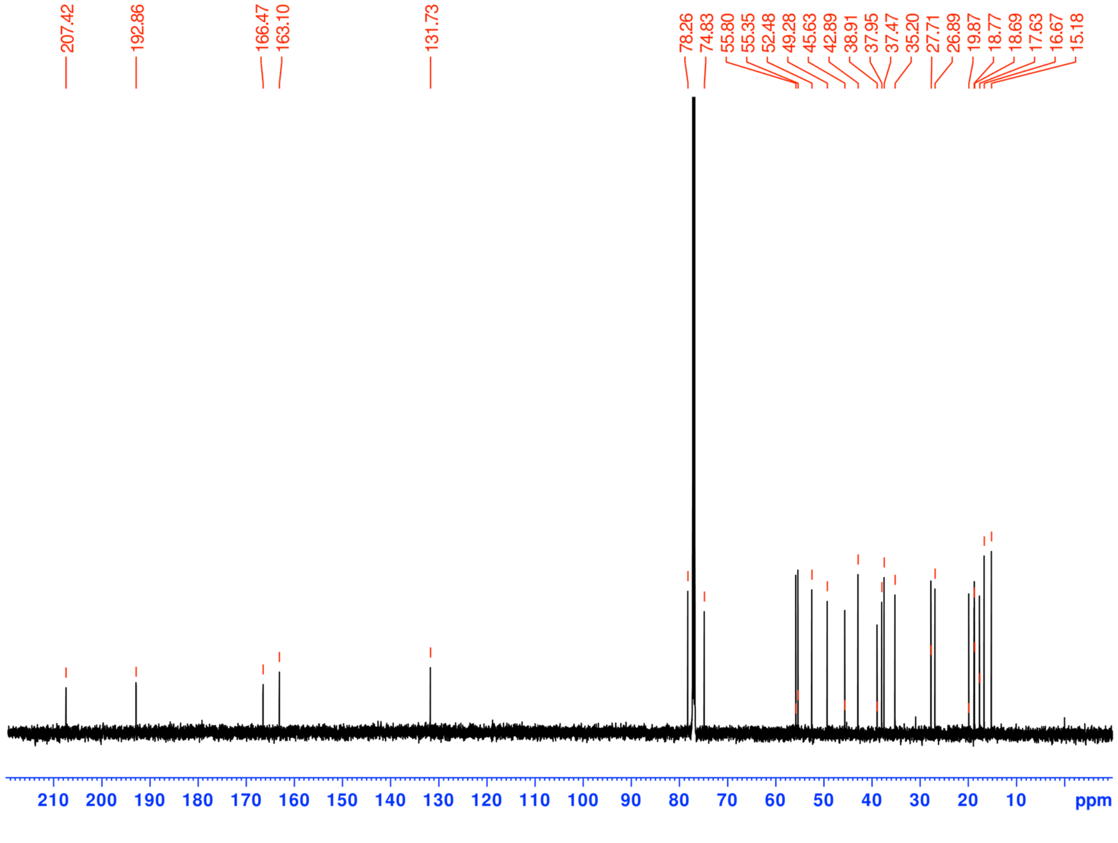

Figure S20. ^{13}C NMR spectrum of **5** in $CDCl_3$ at 150 MHz.

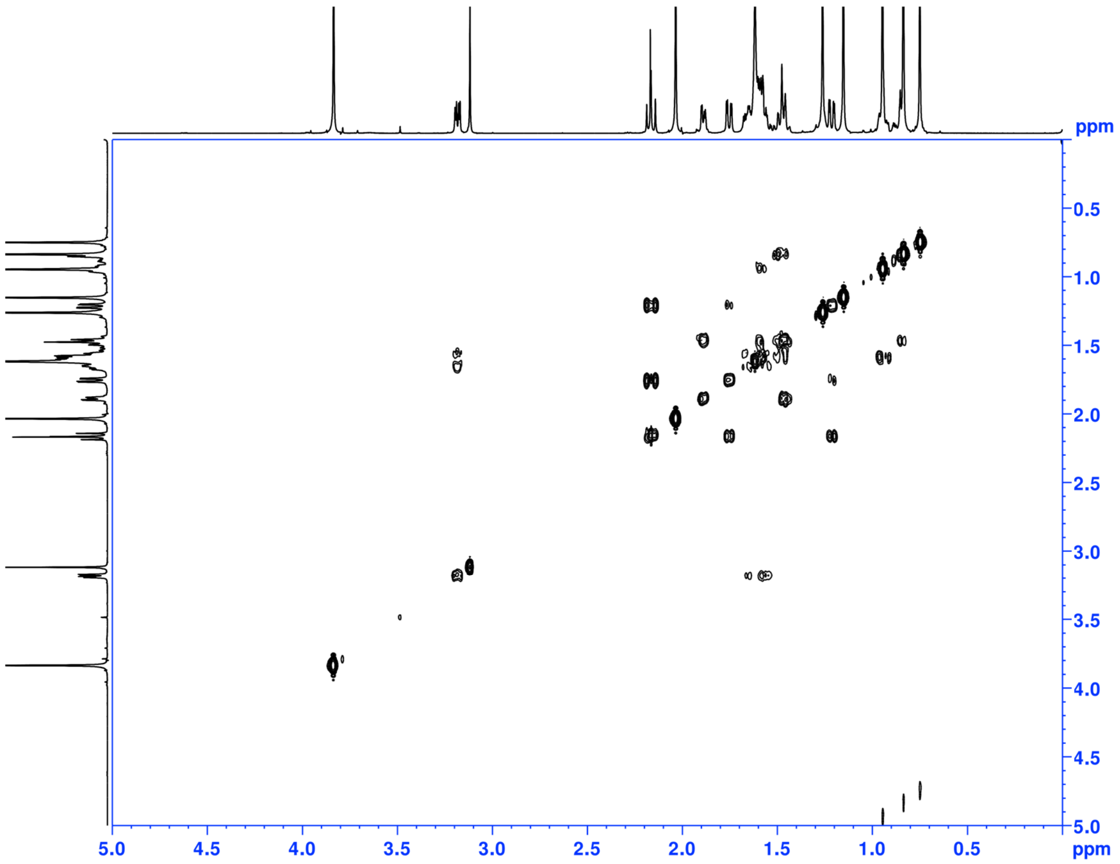

Figure S21. ^{1}H-^{1}H COSY spectrum of **5** in $CDCl_3$.

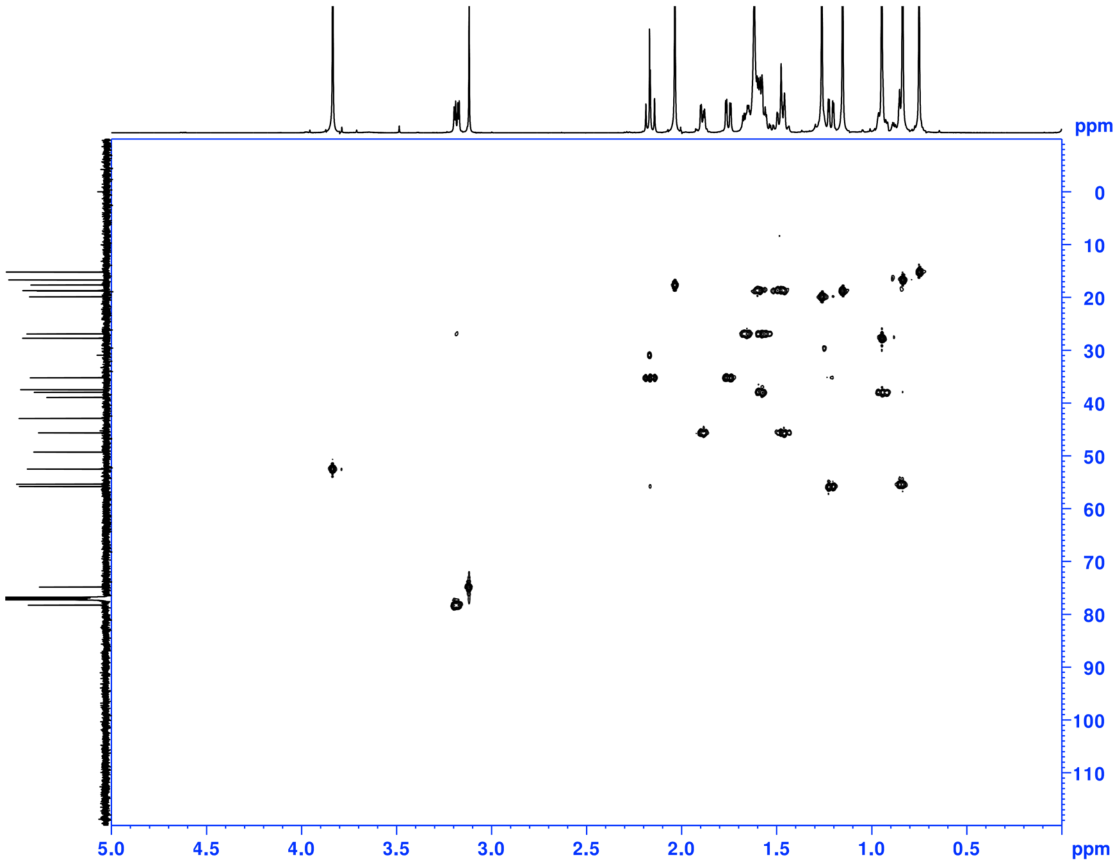

Figure S22. HSQC spectrum of **5** in $CDCl_3$.

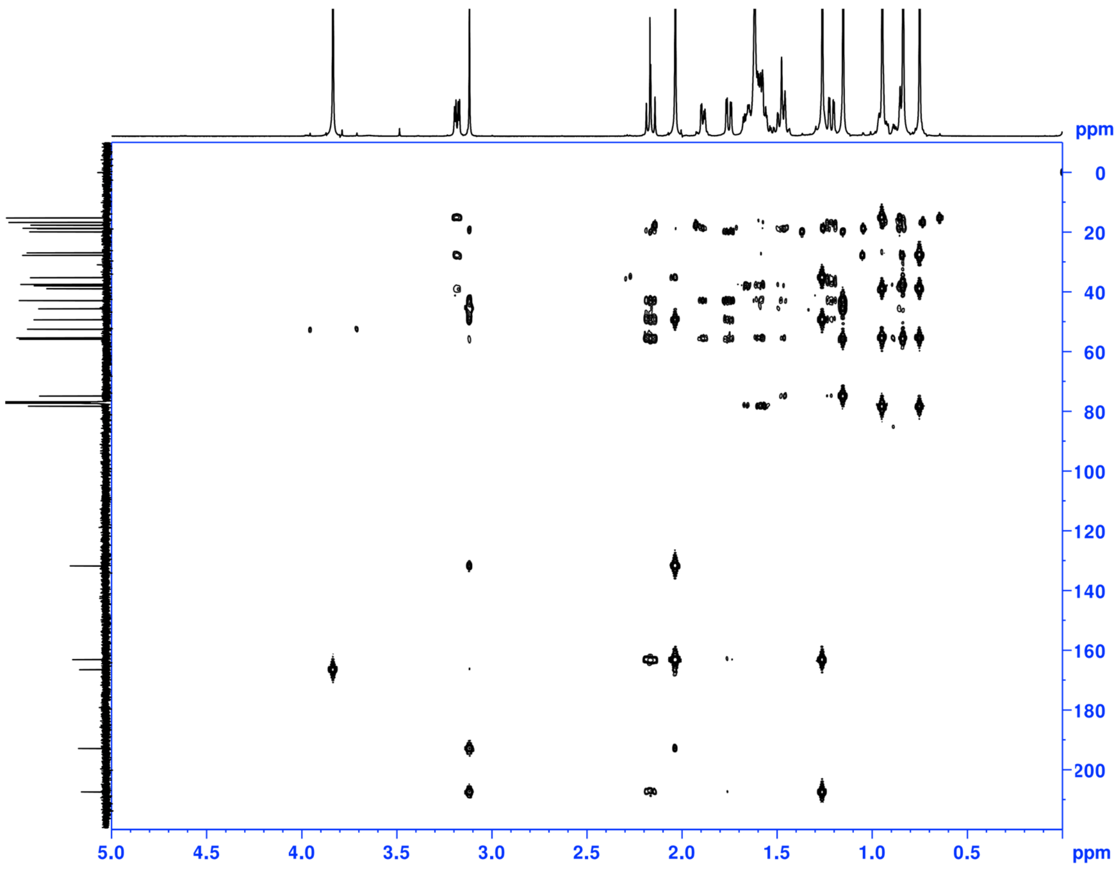

Figure S23. HMBC spectrum of **5** in $CDCl_3$.

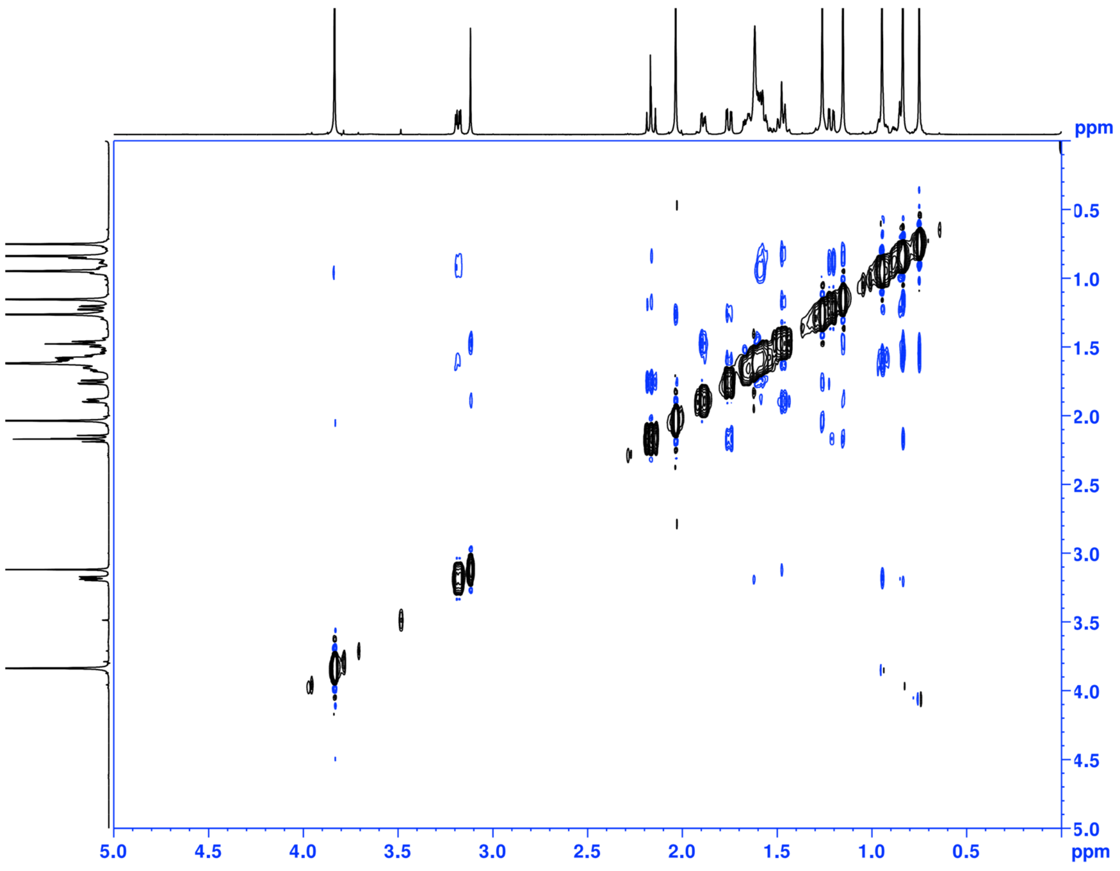

Figure S24. NOESY spectrum of **5** in $CDCl_3$.

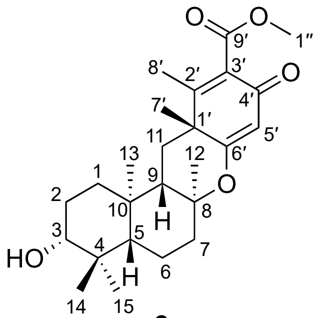

6

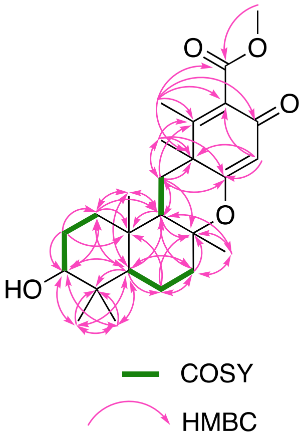

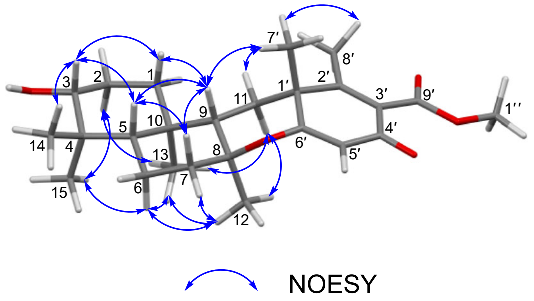

position	δ_C, type	δ_H, mult. (J in Hz)
1	37.3, CH_2	1.68 (α), ddd (15.2, 6.9, 3.5)
		1.10 (β), td (13.2, 3.5)
2	27.0, CH_2	1.63 (α), qd (12.2, 3.5)
		1.74 (β), m
3	78.5, CH	3.28, dd (11.6, 4.5)
4	38.9, C	
5	55.3, CH	1.05, dd (12.0, 2.0)
6	19.6, CH_2	1.39 (α), qd (13.1, 3.1)
		1.83 (β), dq (14.2, 3.2)
7	40.5, CH_2	1.99 (α), dt (12.5, 3.2)
		1.72 (β), m
8	84.4, C	
9	51.4, CH	1.89, dd (13.2, 2.8)
10	36.8, C	
11	28.5, CH_2	1.54 (α), t (13.0)
		1.79 (β), dd (12.9, 2.9)
12	21.6, CH_3	1.15, s
13	15.7, CH_3	0.77, s
14	28.0, CH_3	1.03, s
15	15.2, CH_3	0.79, s
1′	42.1, C	
2′	158.0, C	
3′	132.5, C	
4′	183.6, C	
5′	113.2, CH	5.78, s
6′	177.6, C	
7′	27.5, CH_3	1.46, s
8′	15.5, CH_3	1.96, s
9′	167.7, C	
1″	52.3, CH_3	3.87, s

^{1}H NMR: 600 MHz, ^{13}C NMR: 150 MHz (in $CDCl_3$)

Figure S25. NMR data of 5′-desmethylinsuetusin B1 (**6**).

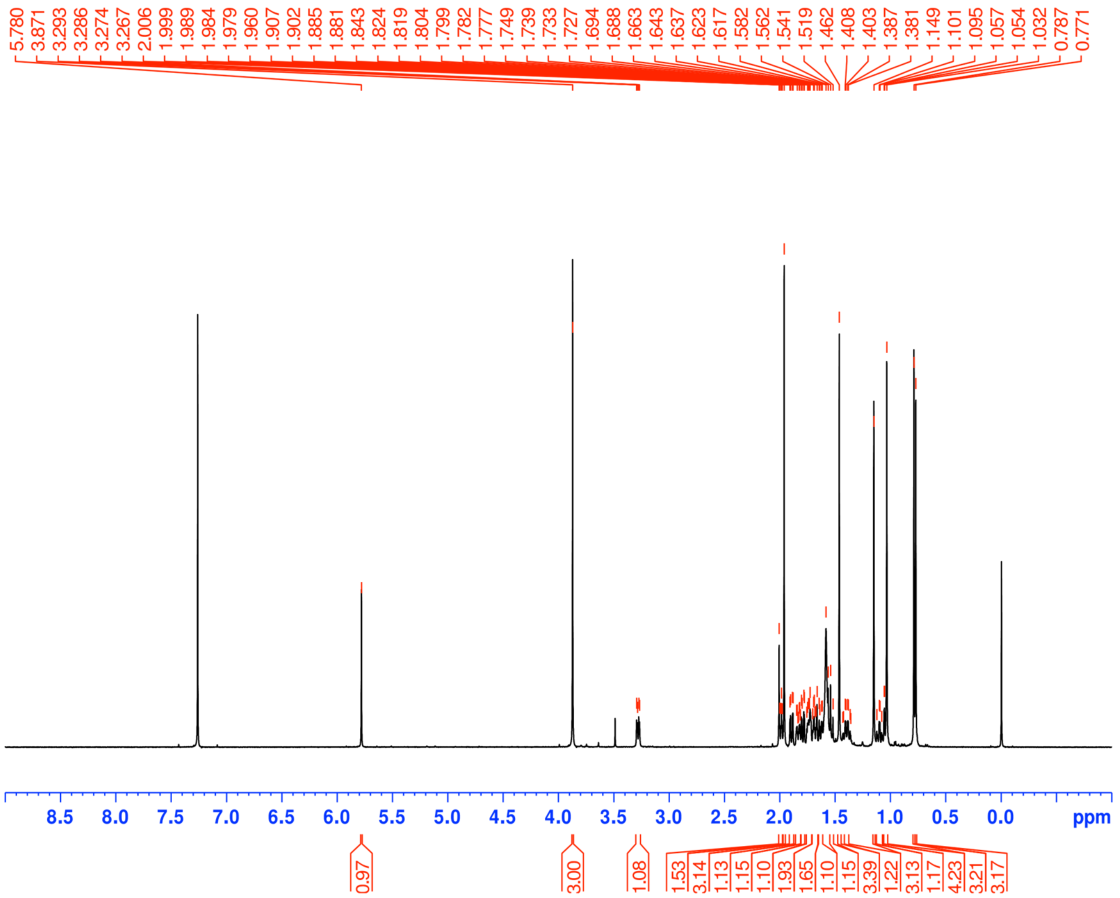

Figure S26. ^{1}H NMR spectrum of 5′-desmethylinsuetusin B1 (**6**) in $CDCl_3$ at 600 MHz.

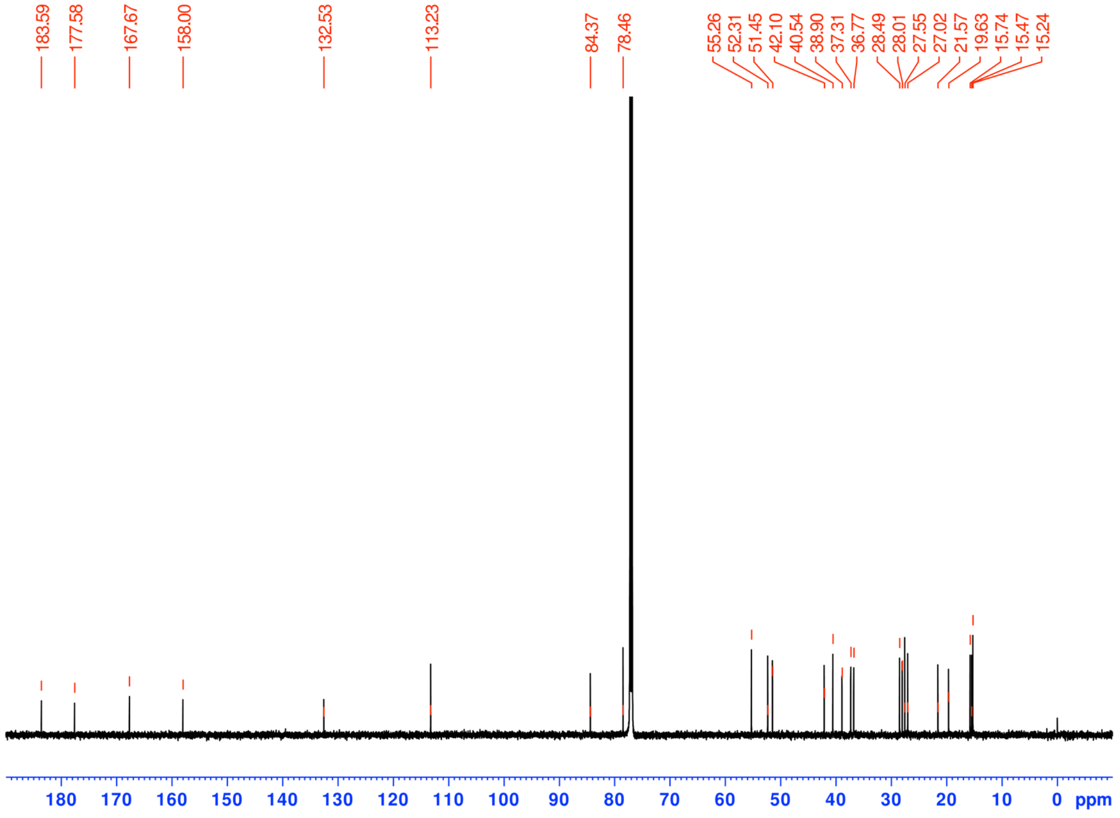

Figure S27. ^{13}C NMR spectrum of 5′-desmethylinsuetusin B1 (**6**) in $CDCl_3$ at 150 MHz.

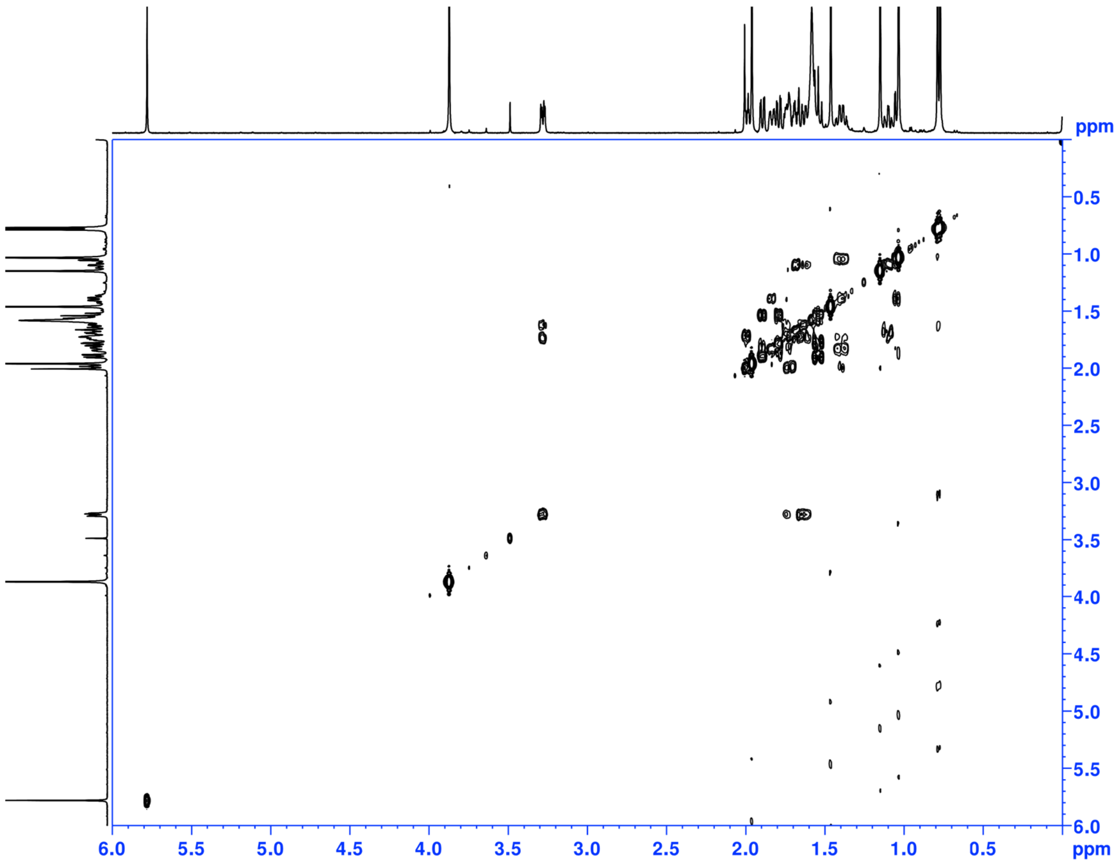

Figure S28. ^{1}H-^{1}H COSY spectrum of 5′-desmethylinsuetusin B1 (**6**) in $CDCl_3$.

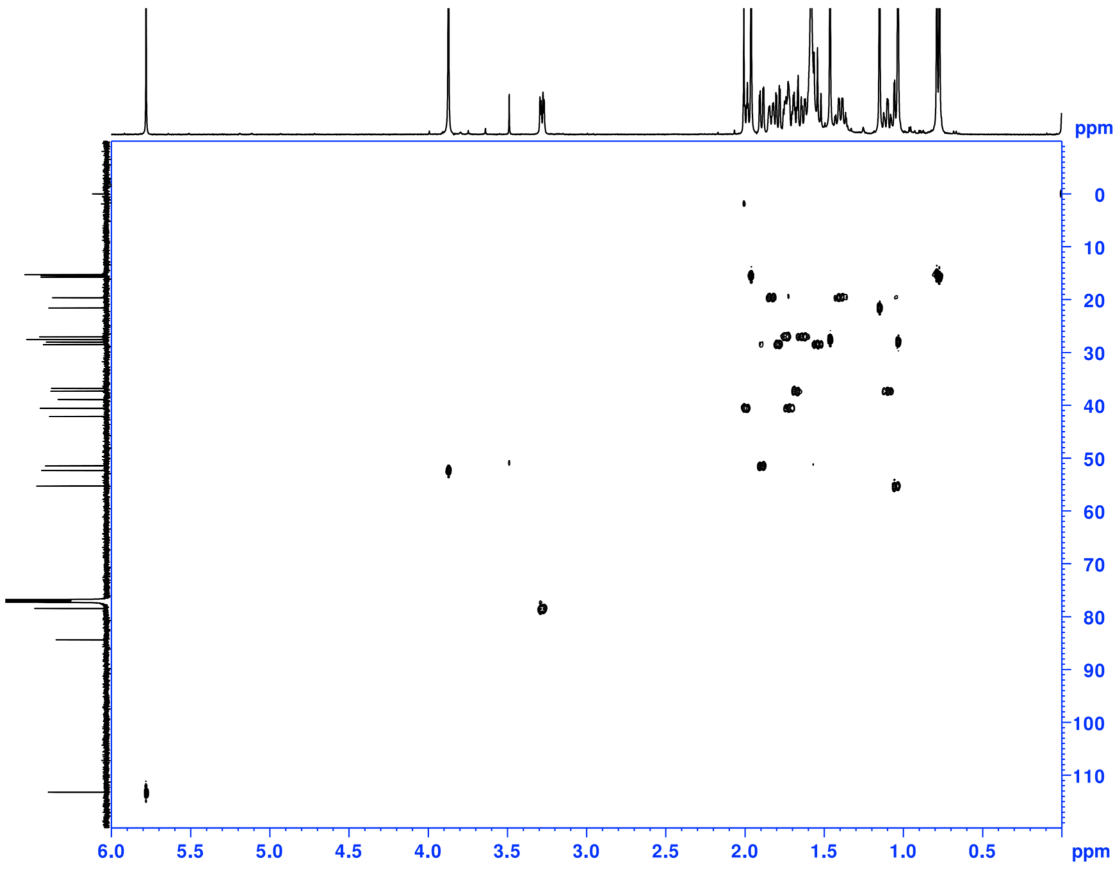

Figure S29. HSQC spectrum of 5′-desmethylinsuetusin B1 (**6**) in $CDCl_3$.

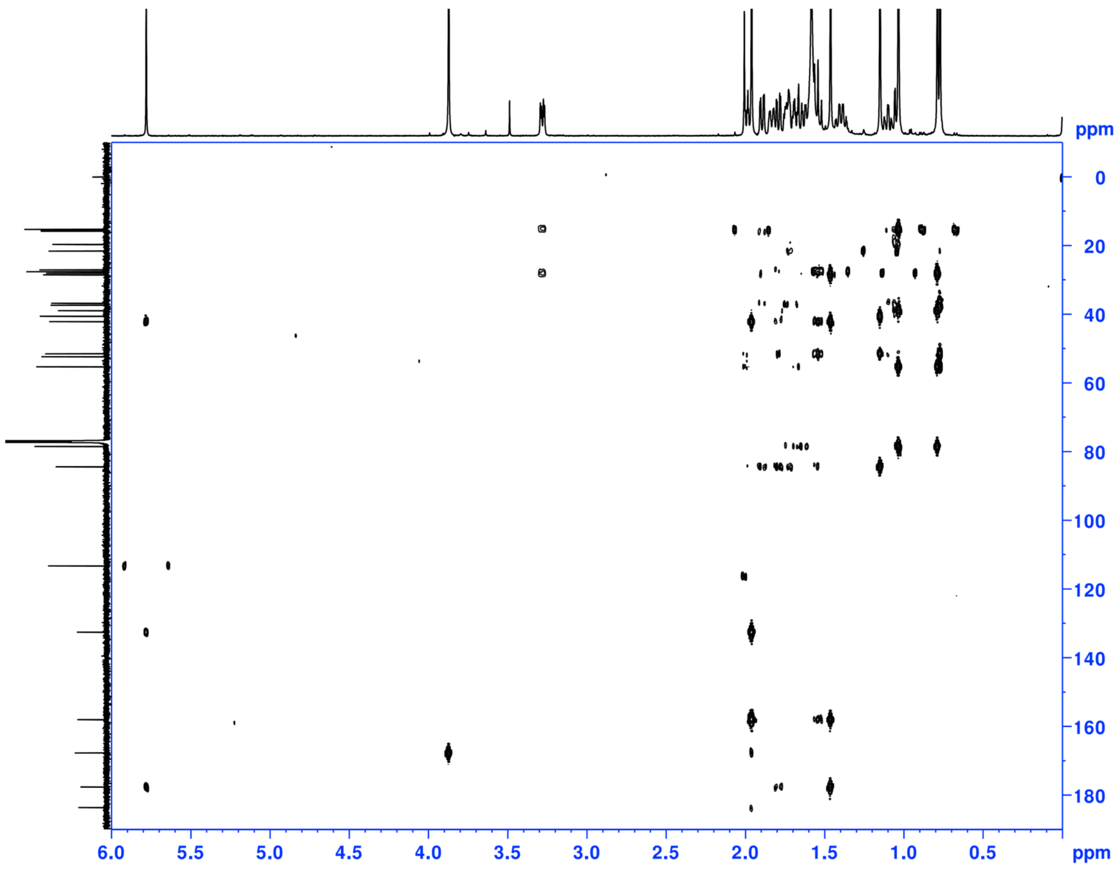

Figure S30. HMBC spectrum of 5′-desmethylinsuetusin B1 (**6**) in $CDCl_3$.

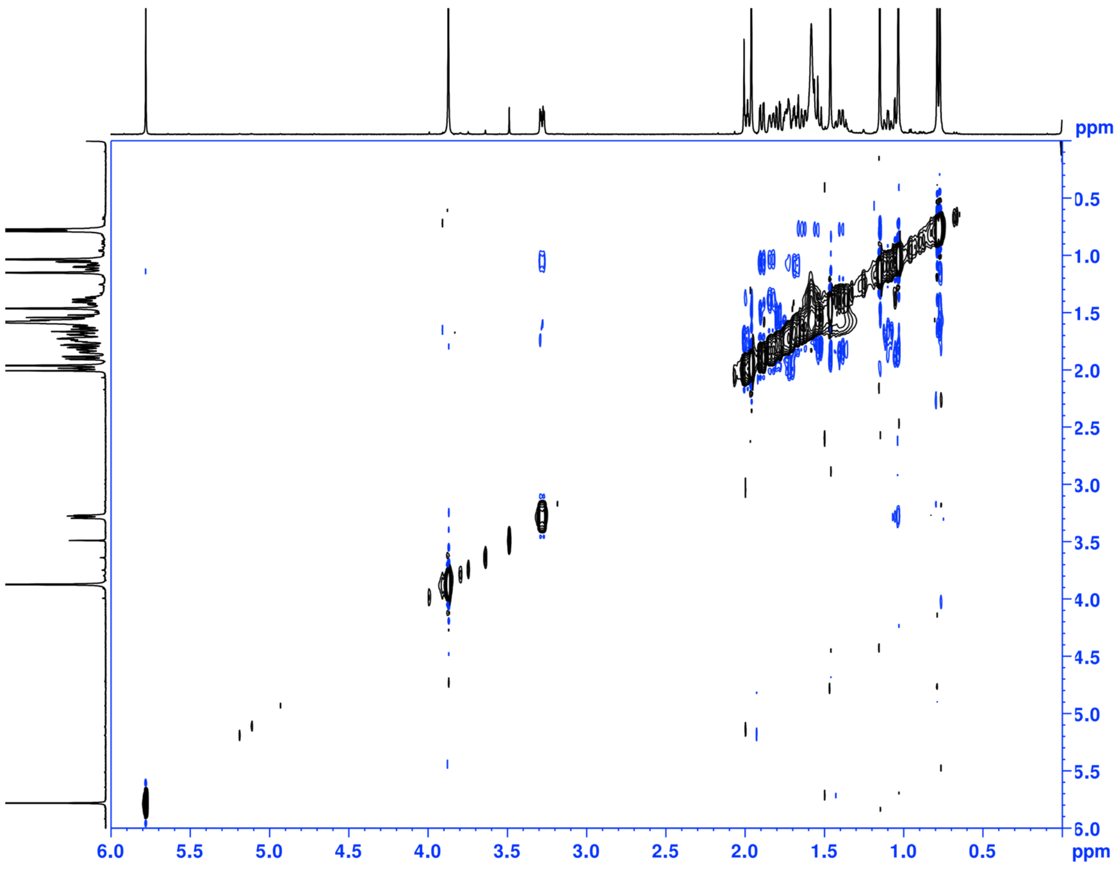

Figure S31. NOESY spectrum of 5′-desmethylinsuetusin B1 (**6**) in $CDCl_3$.

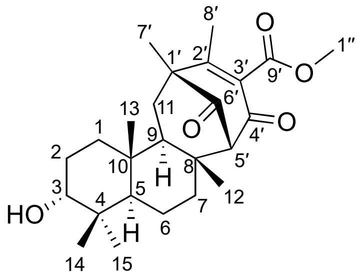

7

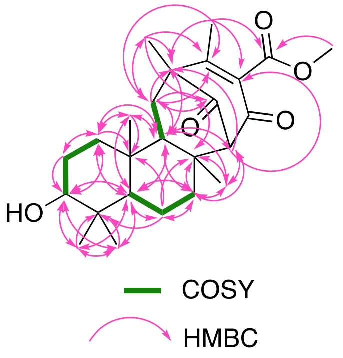

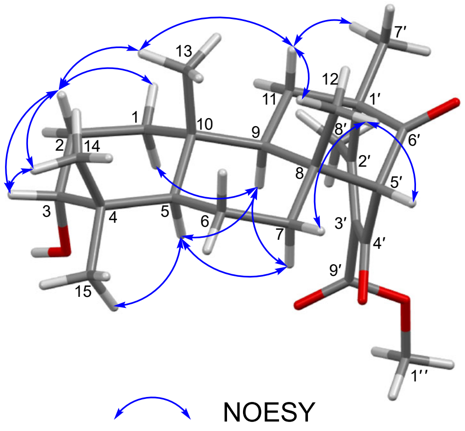

position	δ_C, type	δ_H, mult. (J in Hz)
1	32.9, CH_2	1.28 (α), m
		1.32 (β), m
2	24.7, CH_2	1.58 (α), dq (14.8, 3.1)
		1.92 (β), dddd (14.8, 12.6, 4.3, 2.4)
3	76.0, CH	3.41, t (2.6)
4	37.5, C	
5	48.8, CH	1.30, m
6	18.0, CH_2	1.47, m
7	36.8, CH_2	1.65 (α), td (13.7, 2.5)
		1.46 (β), m
8	44.5, C	
9	49.4, CH	1.46, m
10	37.0, C	
11	34.0, CH_2	1.81 (α), dd (13.7, 3.6)
		1.63 (β), t (13.4)
12	21.0, CH_3	0.96, s
13	16.9, CH_3	0.88, s
14	21.9, CH_3	0.83, s
15	28.0, CH_3	0.93, s
1′	52.4, C	
2′	160.5, C	
3′	137.2, C	
4′	192.8, C	
5′	75.8, CH	2.79, s
6′	205.8, C	
7′	18.5, CH_3	1.31, s
8′	16.9, CH_3	1.96, s
9′	166.7, C	
1″	52.6, CH_3	3.86, s

^{1}H NMR: 600 MHz, ^{13}C NMR: 150 MHz (in $CDCl_3$)

Figure S32. NMR data of 5′-desmethylinsuetusin A1 (**7**).

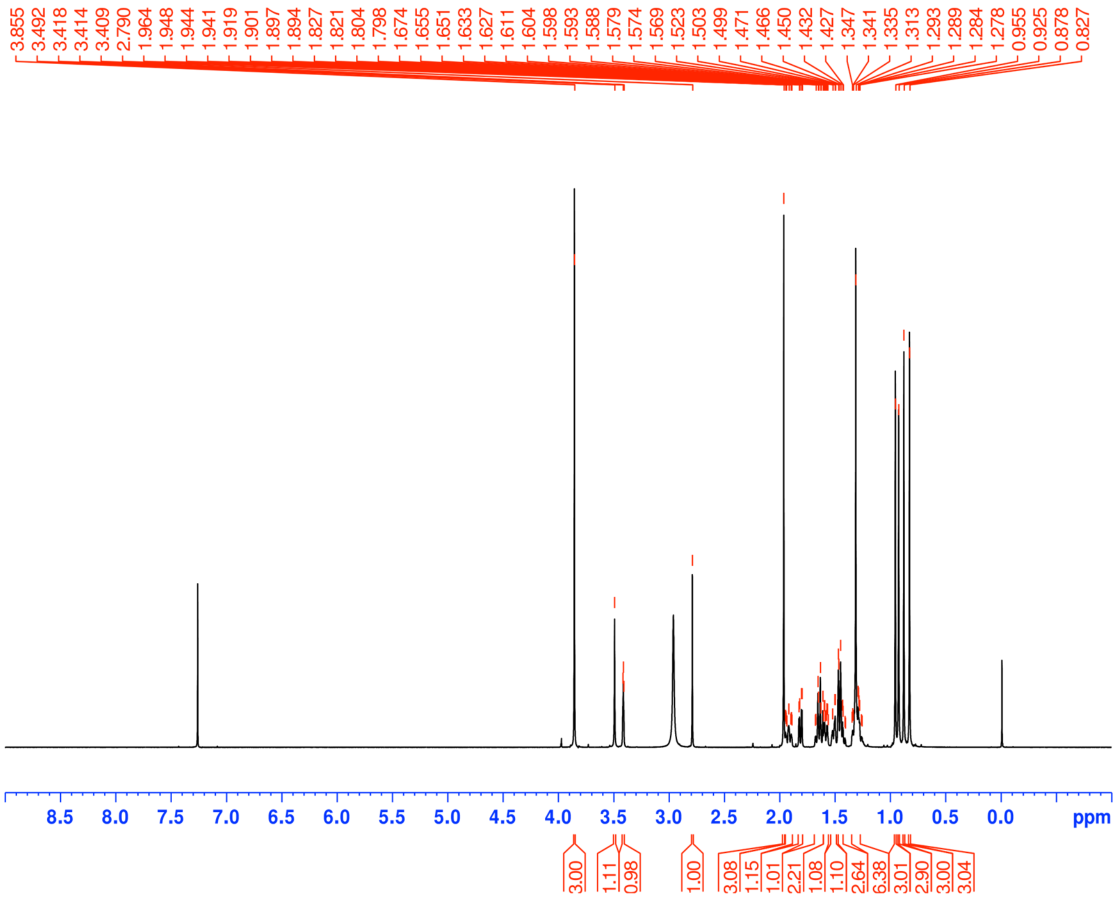

Figure S33. ^{1}H NMR spectrum of 5′-desmethylinsuetusin A1 (**7**) in $CDCl_3$ at 600 MHz.

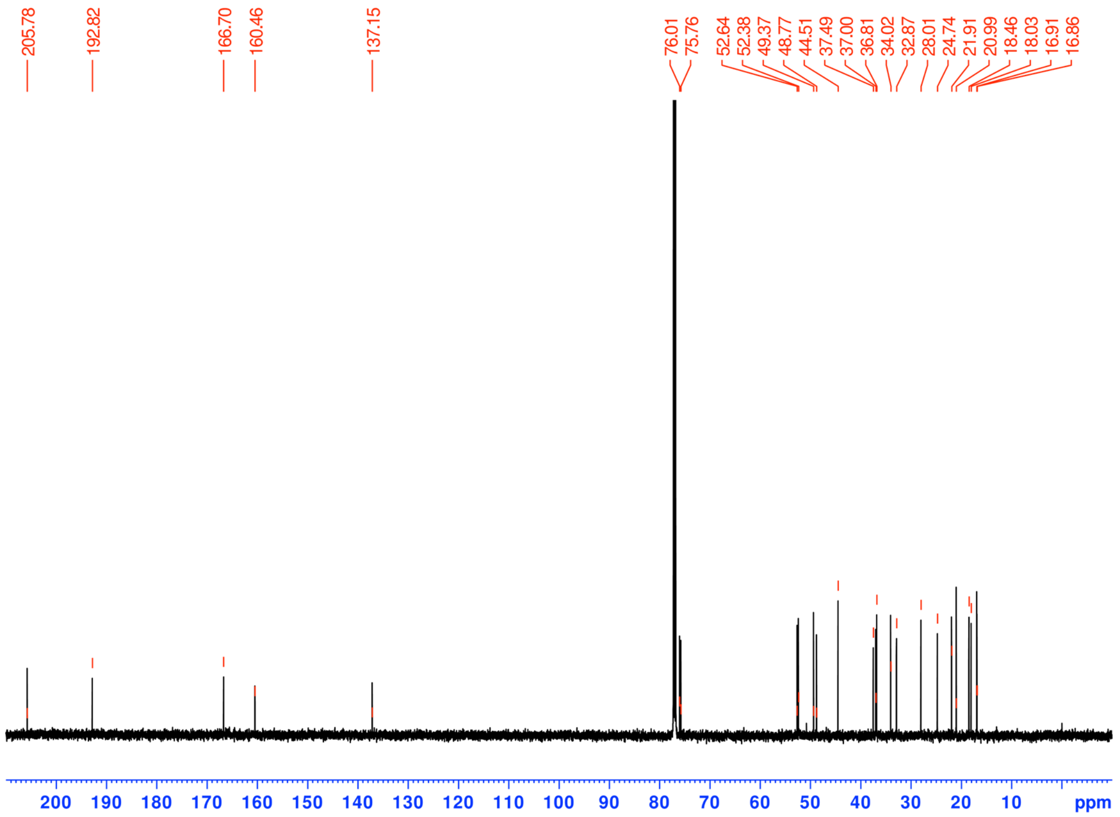

Figure S34. ^{13}C NMR spectrum of 5′-desmethylinsuetusin A1 (**7**) in $CDCl_3$ at 150 MHz.

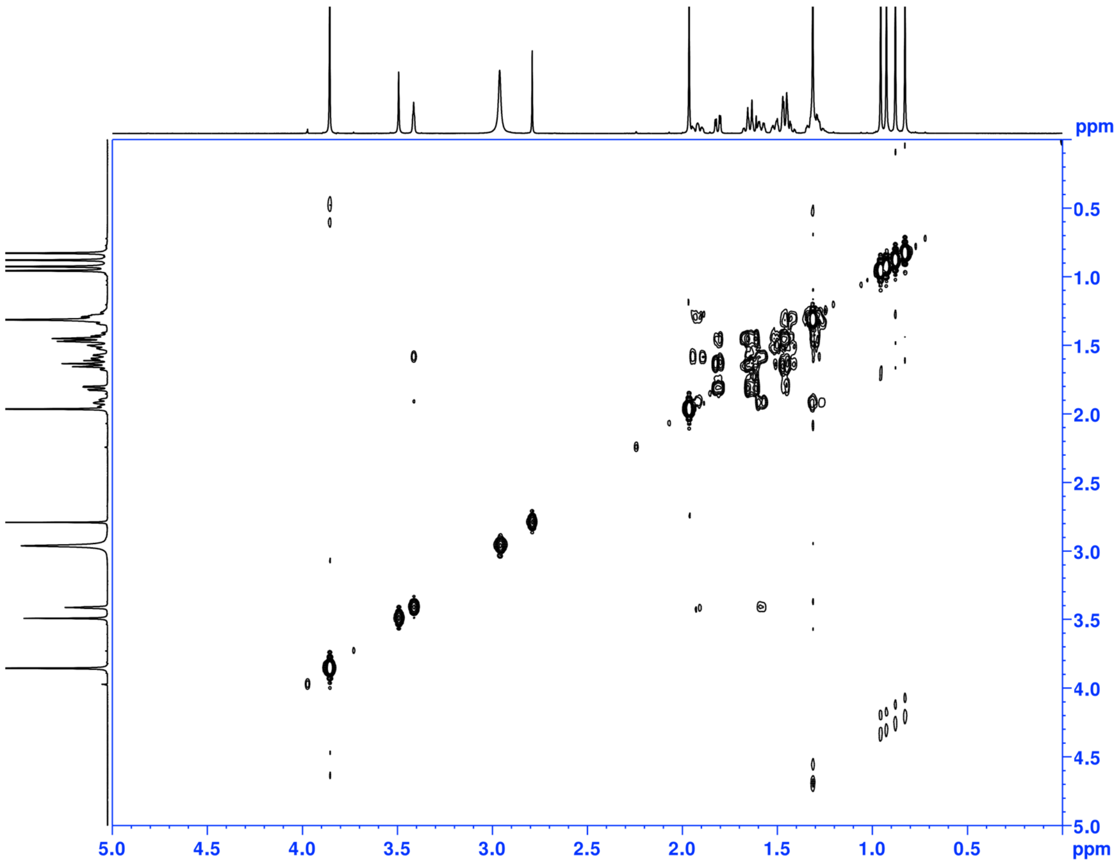

Figure S35. ^{1}H-^{1}H COSY spectrum of 5′-desmethylinsuetusin A1 (**7**) in $CDCl_3$.

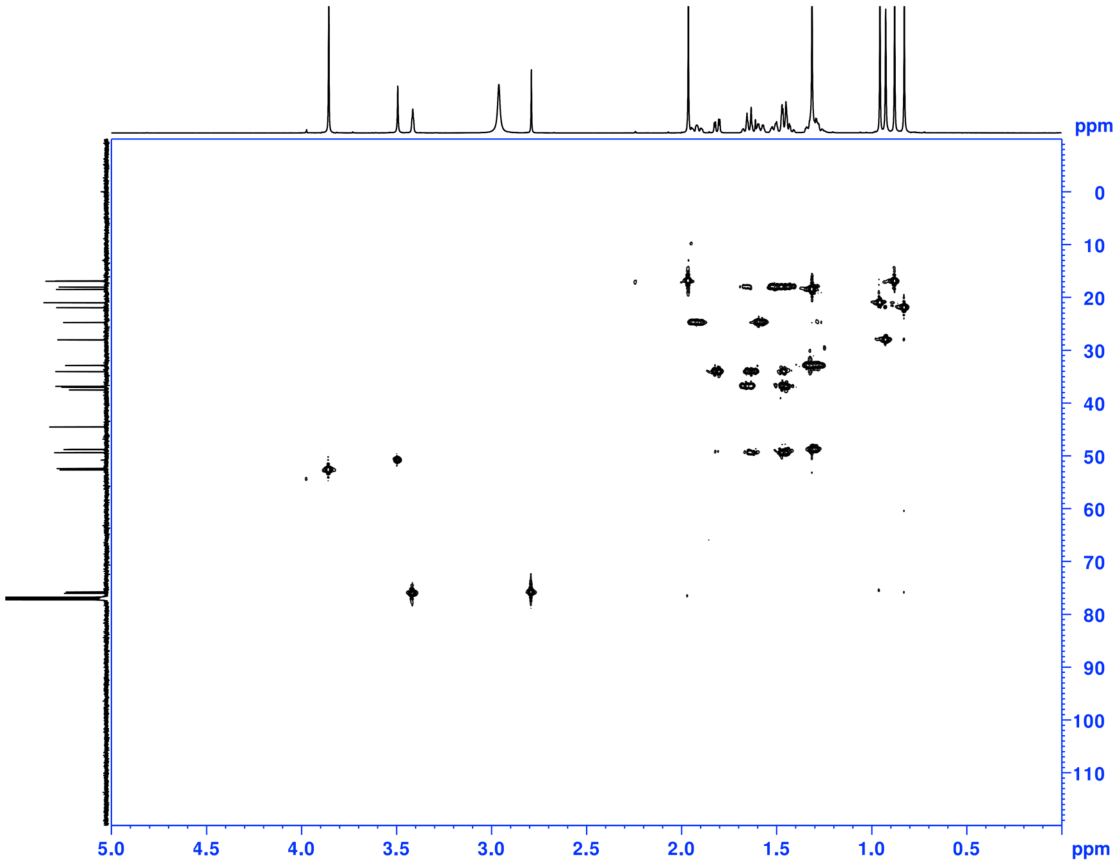

Figure S36. HSQC spectrum of 5′-desmethylinsuetusin A1 (**7**) in $CDCl_3$.

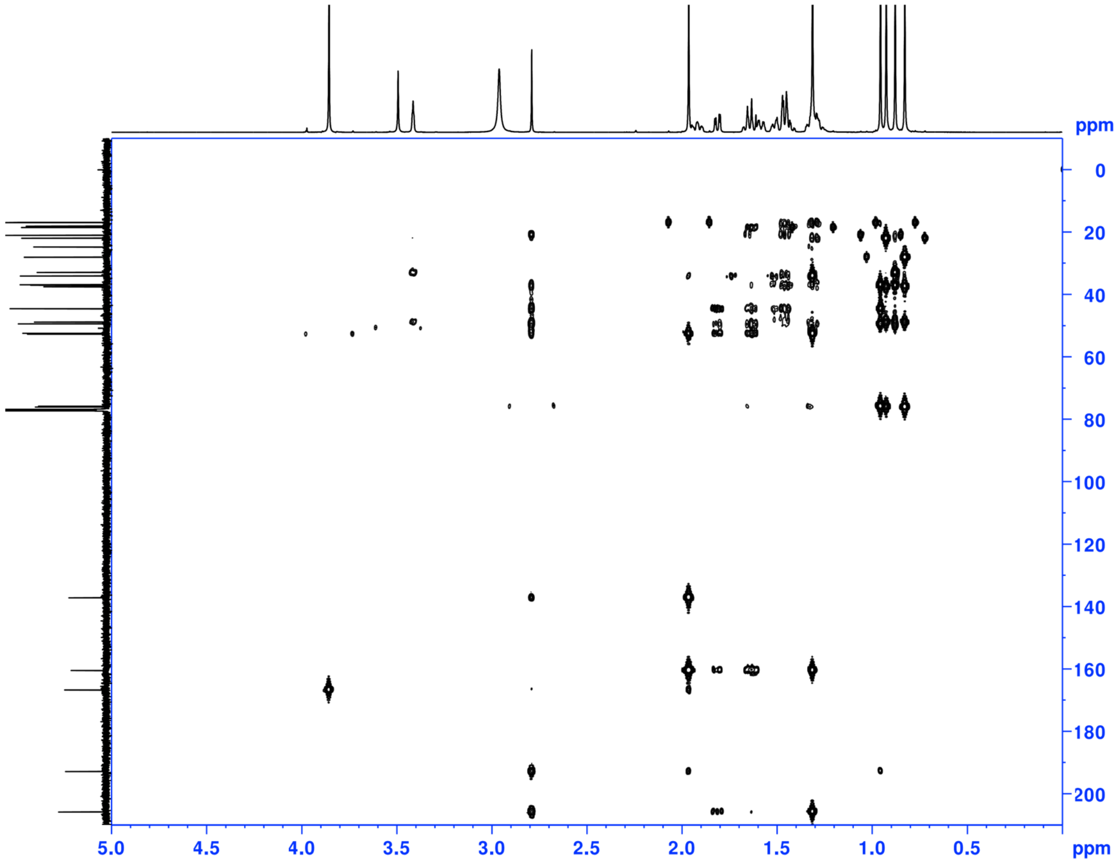

Figure S37. HMBC spectrum of 5′-desmethylinsuetusin A1 (**7**) in $CDCl_3$.

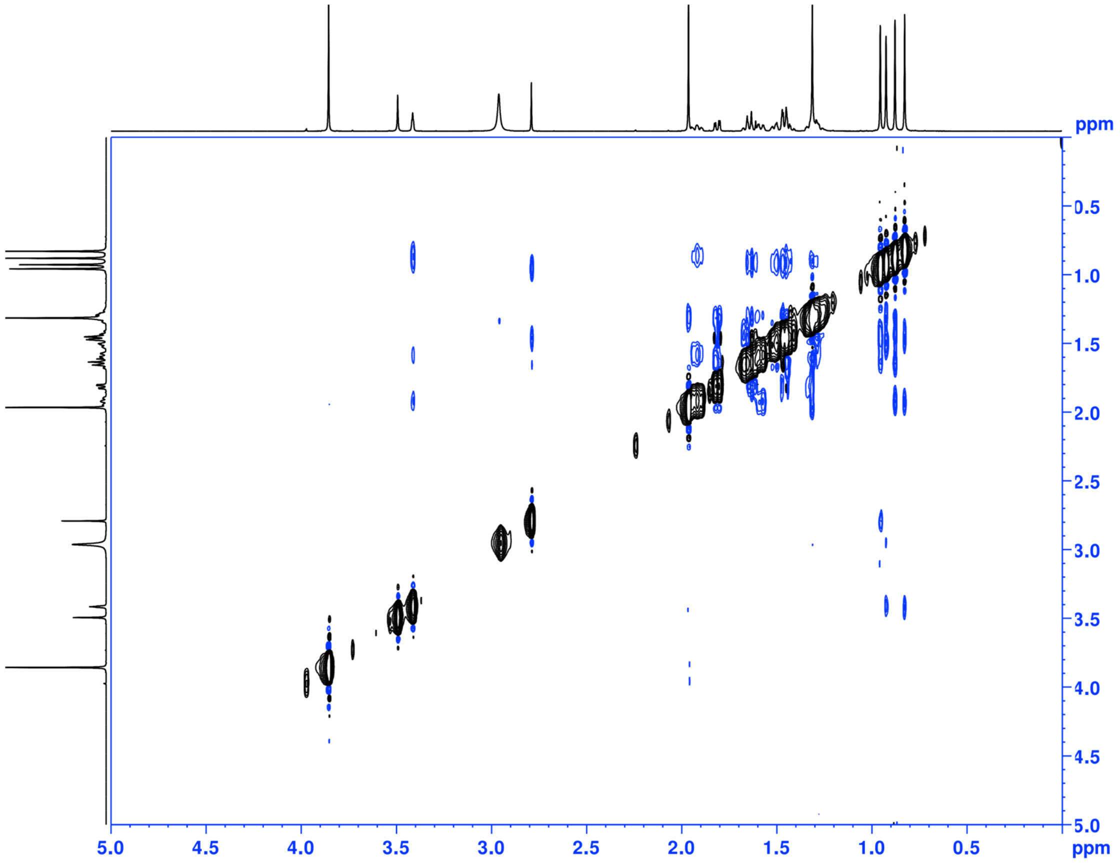

Figure S38. NOESY spectrum of 5′-desmethylinsuetusin A1 (**7**) in $CDCl_3$.

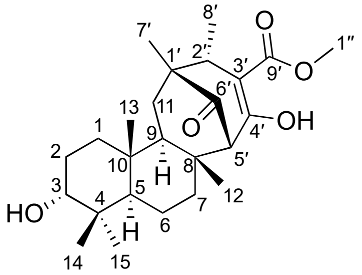

8

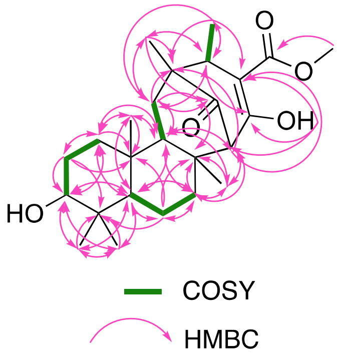

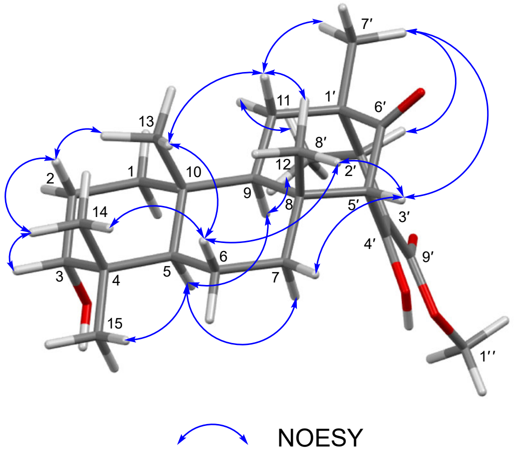

position	δ_C, type	δ_H, mult. (*J* in Hz)
1	32.4, CH_2	1.46, m
2	25.1, CH_2	1.58 (α), brd (14.6)
		1.94 (β), m
3	76.0, CH	3.42, brs
4	37.7, C	
5	49.1, CH	1.34, brd (12.1)
6	18.1, CH_2	1.47, m
7	38.1, CH_2	1.81 (α), td (13.7, 4.3)
		1.48 (β), m
8	44.9, C	
9	48.7, CH	1.64, brd (13.5)
10	37.6, C	
11	33.2, CH_2	1.94 (α), m
		1.29 (β), t (13.7)
12	21.4, CH_3	0.98, s
13	15.5, CH_3	0.81, s
14	22.1, CH_3	0.83, s
15	28.2, CH_3	0.94, s
1′	48.5, C	
2′	38.9, CH	2.52, q (6.8)
3′	105.1, C	
4′	168.5, C	
5′	66.5, CH	2.52, s
6′	211.2, C	
7′	21.7, CH_3	1.06, s
8′	14.5, CH_3	1.25, d (6.8)
9′	172.0, C	
1″	51.7, CH_3	3.79, s
4′-OH		12.41, s

^{1}H NMR: 600 MHz, ^{13}C NMR: 150 MHz (in $CDCl_3$)

Figure S39. NMR data of **8**.

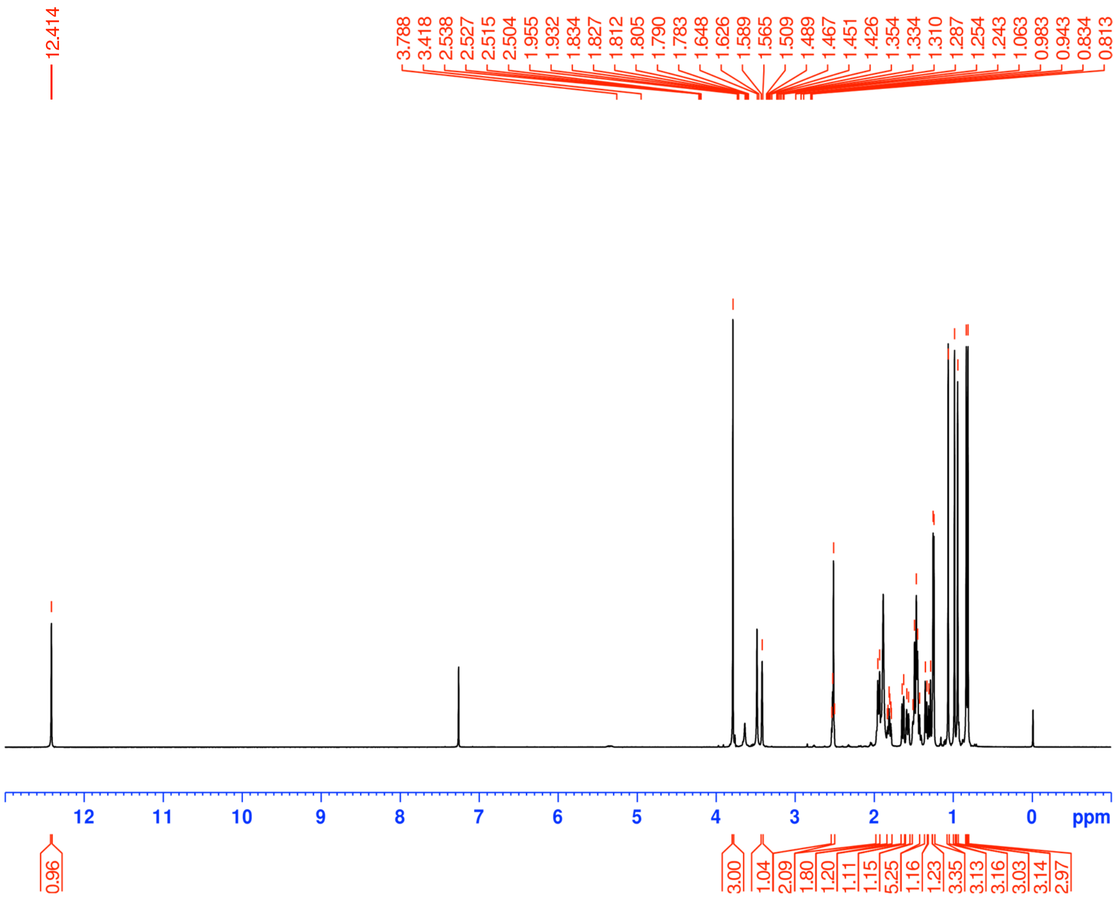

Figure S40. ^{1}H NMR spectrum of **8** in $CDCl_3$ at 600 MHz.

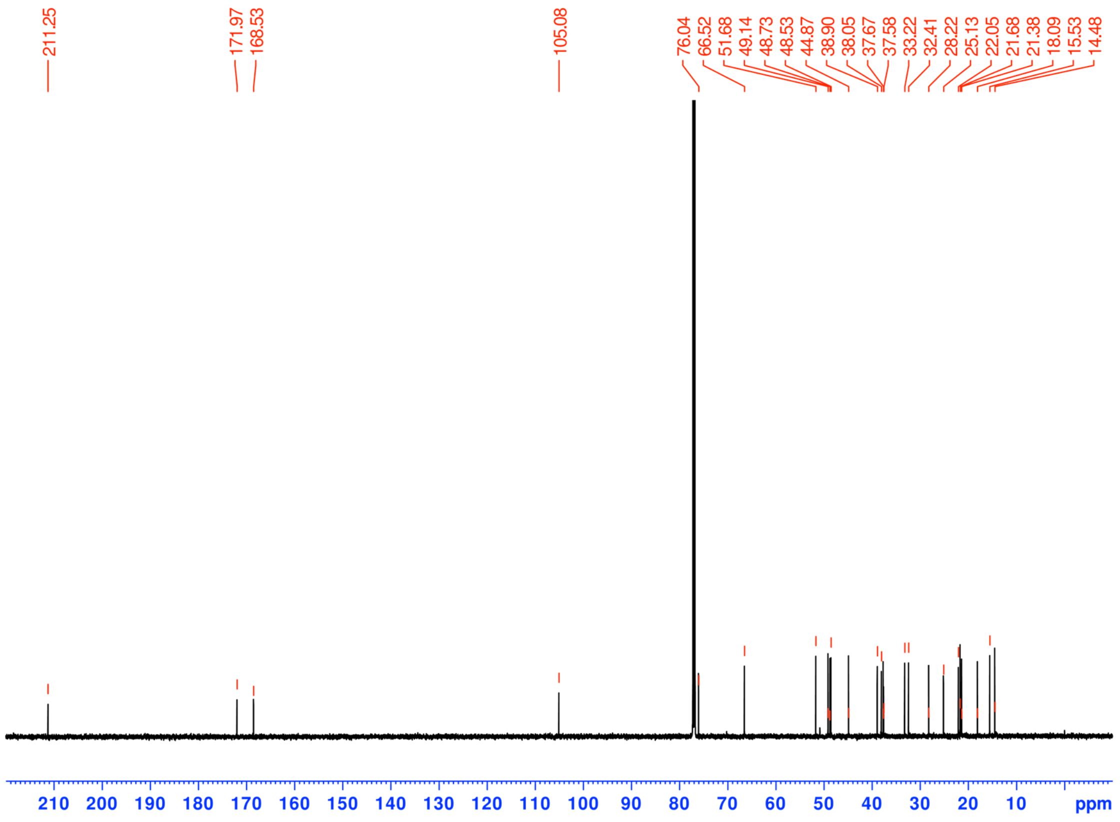

Figure S41. ^{13}C NMR spectrum of **8** in $CDCl_3$ at 150 MHz.

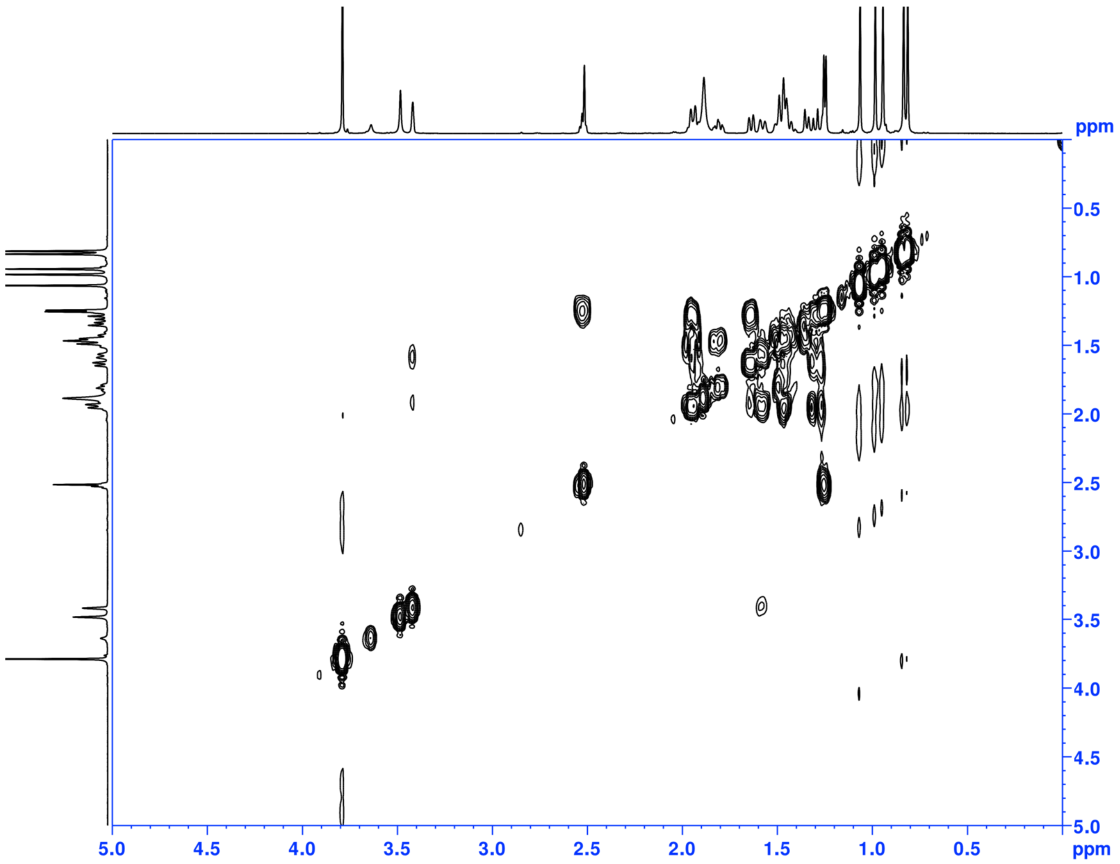

Figure S42. ^{1}H-^{1}H COSY spectrum of **8** in $CDCl_3$.

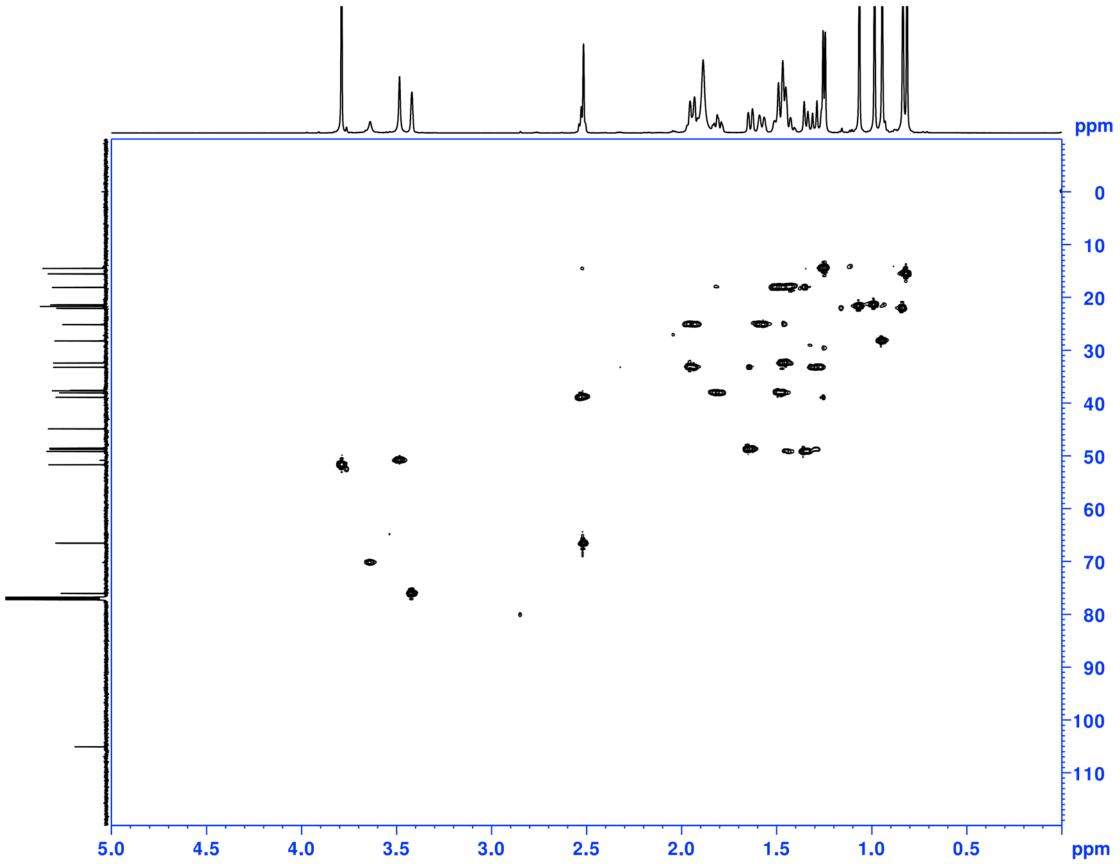

Figure S43. HSQC spectrum of **8** in $CDCl_3$.

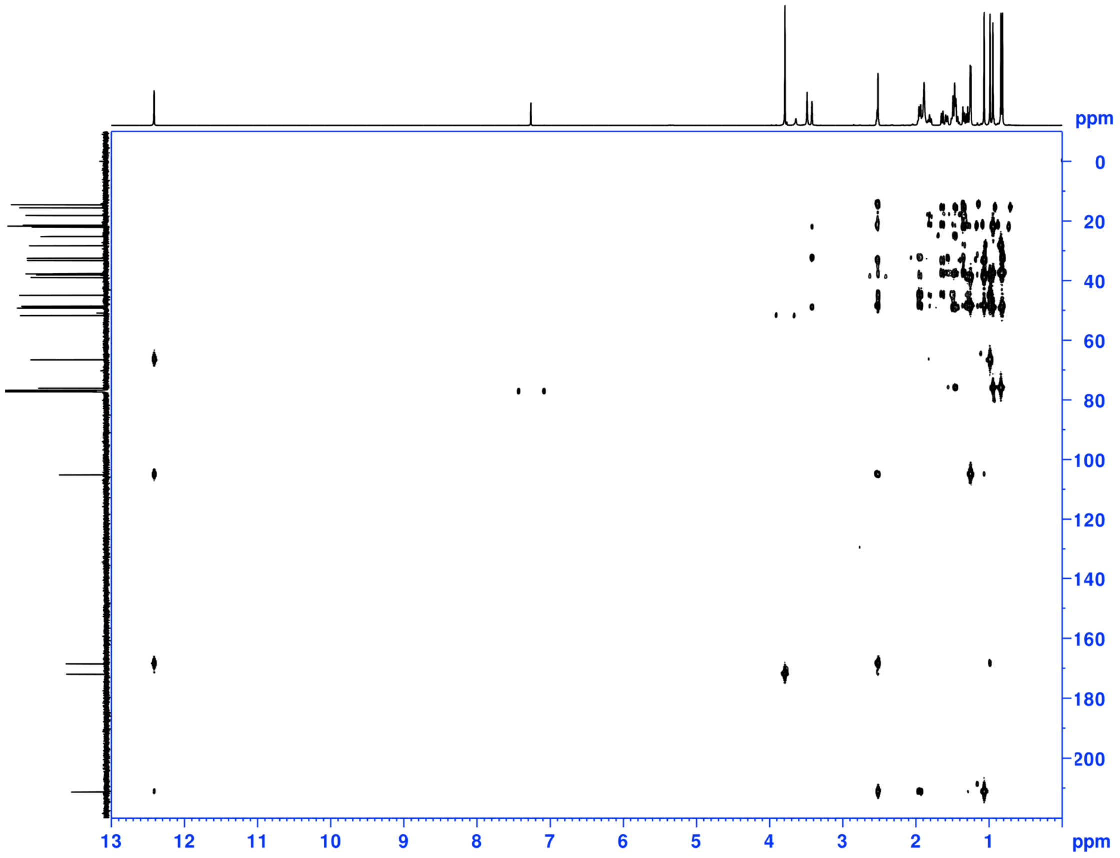

Figure S44. HMBC spectrum of **8** in $CDCl_3$.

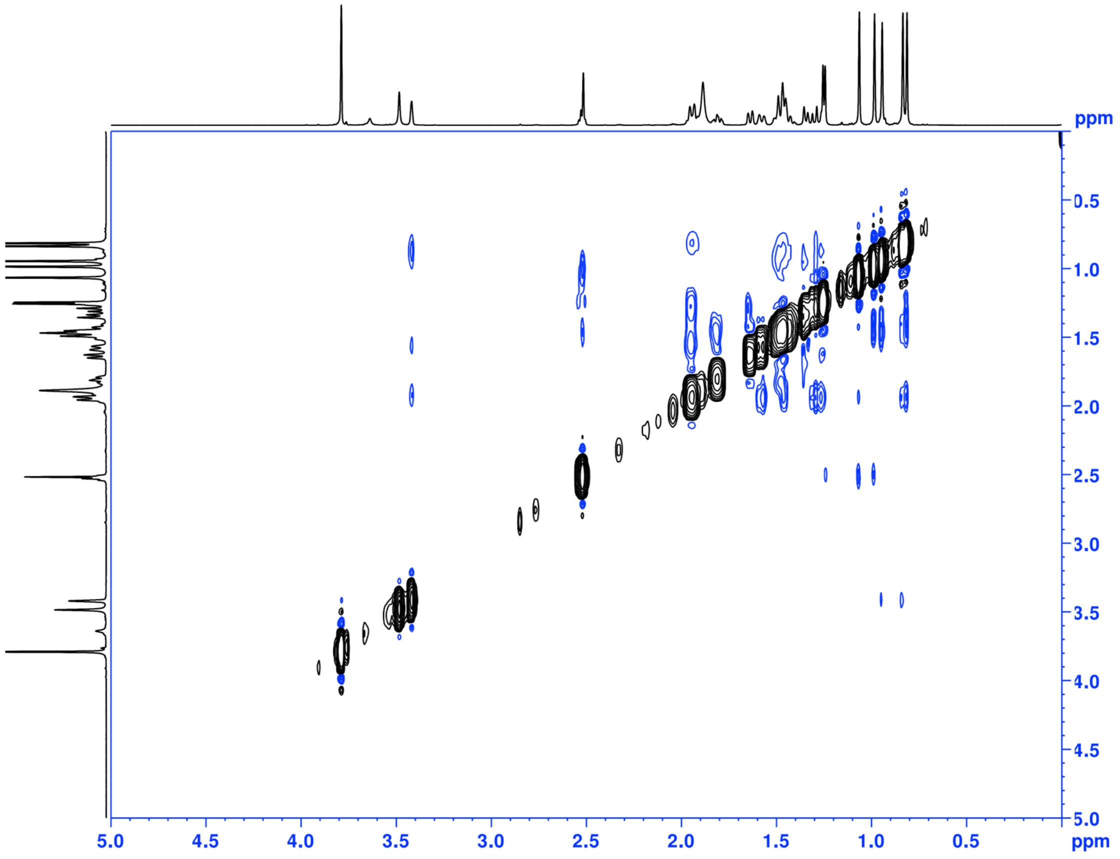

Figure S45. NOESY spectrum of **8** in $CDCl_3$.

Supplementary references

1. Tang, J.; Matsuda, Y. *Chem. Sci.* **2022**, *13*, 10361-10369.

2. Yan, D.; Matsuda, Y. *Org. Lett.* **2021**, *23*, 3211-3215.

3. Matsuda, Y.; Bai, T.; Phippen, C. B. W.; Nødvig, C. S.; Kjærbølling, I.; Vesth, T. C.; Andersen, M. R.; Mortensen, U. H.; Gotfredsen, C. H.; Abe, I.; Larsen, T. O. *Nat. Commun.* **2018**, *9*, 2587.

4. Wei, X.; Matsuyama, T.; Sato, H.; Yan, D.; Chan, P. M.; Miyamoto, K.; Uchiyama, M.; Matsuda, Y. *J. Am. Chem. Soc.* **2021**, *143*, 17708-17715.

5. Chen, L.; Tang, J.-W.; Liu, Y. Y.; Matsuda, Y. *Org. Lett.* **2022**, *24*, 4816-4819.

6. Tang, J.; Matsuda, Y. *Angew. Chem. Int. Ed.* **2023**, *62*, e202306046.

7. Matsuda, Y.; Awakawa, T.; Itoh, T.; Wakimoto, T.; Kushiro, T.; Fujii, I.; Ebizuka, Y.; Abe, I. *ChemBioChem* **2012**, *13*, 1738-1741.

8. Farrugia, L. *J. Appl. Crystallogr.* **2012**, *45*, 849-854.
